# Suspended sediment load prediction using sparrow search algorithm-based support vector machine model

**DOI:** 10.1038/s41598-024-63490-1

**Published:** 2024-06-05

**Authors:** Sandeep Samantaray, Abinash Sahoo, Deba Prakash Satapathy, Atheer Y. Oudah, Zaher Mundher Yaseen

**Affiliations:** 1 Department of Civil Engineering, National Institute of Technology Srinagar, Hazratbal, Jammu and Kashmir, 190006 India; 2 Department of Civil Engineering, Odisha University of Technology and Research, Bhubaneswar, Odisha India; 3 Department of Computer Sciences, College of Education for Pure Science, University of Thi-Qar, Nasiriyah, 64001 Iraq; 4 Information and Communication Technology Research Group, Scientific Research Centre, Al-Ayen University, Nasiriyah, Thi-Qar Iraq; 5 Civil and Environmental Engineering Department, King Fahd University of Petroleum & Minerals, 31261 Dhahran, Saudi Arabia

**Keywords:** Sparrow search algorithm, Suspended sediment load, Support vector machine, Brahmani river, Computer science, Civil engineering, Sedimentology, Geomorphology

## Abstract

Prediction of suspended sediment load (SSL) in streams is significant in hydrological modeling and water resources engineering. Development of a consistent and accurate sediment prediction model is highly necessary due to its difficulty and complexity in practice because sediment transportation is vastly non-linear and is governed by several variables like rainfall, strength of flow, and sediment supply. Artificial intelligence (AI) approaches have become prevalent in water resource engineering to solve multifaceted problems like sediment load modelling. The present work proposes a robust model incorporating support vector machine with a novel sparrow search algorithm (SVM-SSA) to compute SSL in Tilga, Jenapur, Jaraikela and Gomlai stations in Brahmani river basin, Odisha State, India. Five different scenarios are considered for model development. Performance assessment of developed model is analyzed on basis of mean absolute error (MAE), root mean squared error (RMSE), determination coefficient (R^2^), and Nash–Sutcliffe efficiency (E_NS_). The outcomes of SVM-SSA model are compared with three hybrid models, namely SVM-BOA (Butterfly optimization algorithm), SVM-GOA (Grasshopper optimization algorithm), SVM-BA (Bat algorithm), and benchmark SVM model. The findings revealed that SVM-SSA model successfully estimates SSL with high accuracy for scenario V with sediment (3-month lag) and discharge (current time-step and 3-month lag) as input than other alternatives with RMSE = 15.5287, MAE = 15.3926, and E_NS_ = 0.96481. The conventional SVM model performed the worst in SSL prediction. Findings of this investigation tend to claim suitability of employed approach to model SSL in rivers precisely and reliably. The prediction model guarantees the precision of the forecasted outcomes while significantly decreasing the computing time expenditure, and the precision satisfies the demands of realistic engineering applications.

## Introduction

### Research background

The movement of suspended sediments is essential in various fields, like water structure designs, water management, and dam and river engineering^1,2^. Modelling the quantity of sediment load (SL) in rivers is vital for designing flow control and water storage facilities, for example, canals and dams^3,4^. In addition, suspended sediments impact drinking water quality supplies of residential localities and water requirements of industry and agriculture^5,6^. SSL is the outcome of various physical procedures, comprising detachment, transport, and settlement of particles that depend upon intensity and magnitude of rainfall, discharge in river network, land use, physical features of soil, and topography. Several studies have reported rainfall and discharge as the key governing aspects for SSL^7–9^. Human activities like deforestation and land-use change can upsurge input of fine sediment to streams^10,11^. In this context, SSL in river networks can be defined as collective outcome of catchment management applications^12^. This suggests that suspended sediments result from rivers' complex and non-linear flow processes^13^. Hence, modelling the non-linear relationship amid river flow and suspended sediments utilizing different non-linear approaches has become a fundamental challenge for various scientific communities, such as engineering and water resources management^14–17^.

Because of the stochastic aspect of sediment particle transport in the flow and the non-linear behavior of suspended sediment problems, conventional computational techniques may be unsuccessful for precise SSL predictions^18,19^. In this context, AI methodologies have commonly been employed for predicting SSL in rivers^20,21^. Compared to mathematical and physical approaches, machine learning (ML) techniques are more prevalent because of lower cost, few parameters, and high accuracy^22^. ML algorithms have been effectively employed in the last two decades to model various hydrological and water resources problems. Employed ML algorithms for SSL prediction can be categorized into standalone and hybrid techniques. Taking examples of the implementation of standalone methods, various researchers investigated the efficacy of the ANN model for SL prediction^23–25^. In another study, SVM model was applied for predicting SL in three rivers in Malaysia. Outcomes revealed that predictive performance of SVM was higher compared to conventional techniques^26^. Again, regression model, GEP, and ANFIS (adaptive neuro-fuzzy inference system) methods were developed for SL prediction in three Malaysian rivers. They found that performance of GEP model was best compared to other models^27^. Potential of ANNs, ANFIS, WANN, and customary sediment rating curve (SRC) models were studied for daily SSL estimation in two gauging sites in USA^28^. Estimation accuracy assessment of applied models presented that WANN provided more precise SSL estimations than other models. Long short-term memory (LSTM) was considered for estimating sediment concentration on a daily basis in Schuylkill River, United States^29^. Also, Linear Regression (LR), MLP, Extreme Gradient Boosting, and LSTM were applied for sediment load prediction with different time intervals^30^. Both studies revealed that LSTM performed better than other applied models. In another study, ANN, ANFIS, least square-SVM (LS-SVM), and group method of data handling (GMDH) were employed to estimate SSL using monthly sediment and average river flow data^31^. Outcomes revealed that LS-SVM model generated higher accuracies compared to other models. Kumar and Tripathi^32^ predicted SSC applying ANN, SVM, and MLR models using runoff and sediment data from Musiri gauge station, located on bank of River Cauvery, India. Their findings showed that ANN model with a solitary hidden layer is most appropriate for SSC predictions.

### Problem statement and literature

Given the importance of sediment load movement in sculpting the Earth's surface, the challenges associated with sediment transport prediction have attracted a great deal of interest^33^. The important thing is usually to use a tool that is well-founded in order to assess the suspended sediment load. Even with the advancement of modern numerical models, the movement of river sediment load is still difficult to understand. For example, a direct technique that necessitates the installation of a hydrometric station for sample collection and monitoring can be expensive and time-consuming, particularly in remote locations^34^. Although indirect approaches are less costly, the susceptibility of sediment particles to various environmental variables makes it difficult to reconcile theoretical results with observations^35^. Furthermore, a limited range of environment circumstances are covered by the majority of experimentally validated equations, thereby limiting their applicability^36^. Alternatively, the emergence of artificially intelligent algorithms, including ANN and SVM, has revolutionized many time series forecasting, including silt transport prediction, by avoiding the computation of the complex sediment transport rate. One of the key benefits of this strategy is that it does not require knowledge of the complex sediment transport process's underlying physical mechanism^37^.

Regardless of their broad application, ML algorithms still have various flaws^38–41^. For predicting hydrological variables, it is necessary to train neural network models. Training level finds best values for weight connections, bias values, number of hidden layers, and number of neurons. Conventional training algorithms such as backpropagation algorithms, have a propensity of getting stuck in local minima. In addition, low convergence speed can also hamper the effectiveness of such techniques^42,43^. Even though AI techniques are vastly considered to predict different hydrological variables, such techniques necessitate fine-tuning using training algorithms. The complete AI model must be tuned for completing the ultimate network training. Recently, optimization algorithms like GA, bat algorithm (BA), Grey Wolf Optimization (GWO), shark algorithm (SA), particle swarm optimization (PSO), and firefly algorithm (FA) have been utilized for training soft computing models for determining their best parameter values^44–51^.

Rajaee et al.^52^ applied SRC, multilinear regression (MLR), ANN, and Wavelet-ANN models for daily SSL modelling in Iowa River gauge station (US). They concluded that W-ANN model showed better agreement with collected SSL values and performed superior to other considered models. Kisi et al.^53^ investigated accuracy of ANFIS-GA, ANFIS-PSO, ANFIS-ACO, and ANFIS-BOA models in drought forecasting considering monthly precipitation data of Biarjmand, Ebrahim-Abad, and Abbasabad stations located in Iran. Adnan et al.^54^ proposed an alternative tool named dynamic evolving neural fuzzy inference system (DENFIS) for estimating SSL based on historical sediment and streamflow values recorded at Guangyuan and Beibei stations in China. Obtained results were compared with other two models, and they found that the DENFIS model generated improved SSL predictions. Hassanpour et al.^55^ applied SVR-FCM hybrid model, and compared it with SRC), ANN, ANFIS, and SVR models for predicting daily SSL in River Sistan, Iran. They found that SVR-FCM model predicts SSL more accurately in the specified study region. Banadkooki et al.^42^ proposed hybrid ANN-BA, ANN-PSO, and ANN-ALO models for SSL prediction in Goorganrood basin, Iran. Based on comparison of results, they observed that ANN-ALO model generated most accurate prediction results in the study region. In another study, Ehteram et al.^56^ applied ANN-WA, ANN-PSO, and ANN-BA for optimizing performance of ANN in predicting the rate of SSL accurately in Goorganrood basin, Iran. They found that ANN-WA performed best with accurate SSL predictions. Nhu et al.^57^ used random subspace (RS), SVM-RBF (radial basis function) kernel, random forest (RF), and SVM-NPK (normalized polynomial kernel) for SSL prediction in Haraz catchment situated in mountainous Mazandaran Region (Iran). Obtained results revealed that RS model showed great potential in SL prediction in poor data catchments. PSO, ACO, BA, DE, BOA, and GOA algorithms have been successfully applied recently to improve the prediction accuracy of SVM models; for this reason, BA, GOA, BOA algorithms can be chosen as benchmark optimization methods in this work. Even though these algorithms have produced results that are adequate in the current literature, there is always room to increase the speed at which convergence occurs, find speedy optimal solutions, and prevent tapping in local minima^58^. Therefore, in order to create a reliable predictive model for SSL, a new optimization algorithm sparrow search algorithm is investigated in this work.

### Objective of this study

As discussed in the related literature, optimization algorithms enhance convergence speed of conventional ML models and increase their performance accuracy^48,49,59^. The SSA is a population-based optimization algorithm which was proposed based on foraging and anti-predatory behaviors of sparrow populations, and built upon existing population intelligence algorithms, such as GWO, ALO, and PSO etc. It presents certain advantages in terms of stability, convergence accuracy, and velocity. To the best of the authors' knowledge, no preceding effort has been put into applying an SVM-based SSA model for SSL prediction in Brahmani River basin, hence the objective of present study. By distributing the population of sparrow into three groups: discoverers, entrants, and guards, thresholds and input weights of SVM are optimized. Moreover, various scenarios have been adapted to model input–output architecture for achieving best prediction accuracy for SSL. Lastly, for the performance evaluation between novel SVM-SSA model and other AI algorithms, a complete comparative assessment has been conducted. The outcomes show that proposed SSA algorithm increases convergence speed and efficiently avoids optimization procedure from falling into local optimum.

## Study area

**Figure 1:**
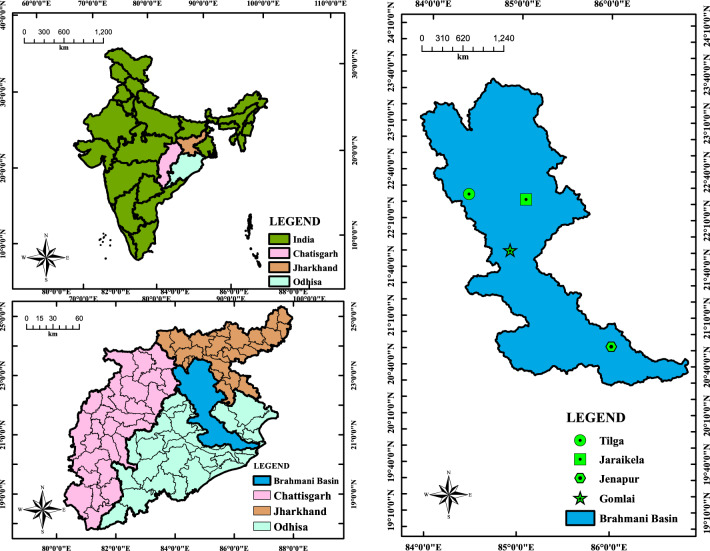
Illustration of Barhmani River basin.

River Brahmani flows in the eastern portion of India between 20° 30′ 10″ to 23° 36′ 42″ N latitudes and 83° 52′ 55″ to 87° 00′ 38″ E longitudes (Fig. 1 “generated using ArcGIS software environment”). On the right of the basin lies the Mahanadi basin, and on the left the Baitarani basin with a total 39,313.50 km^2^ catchment area. Climatic conditions of Brahmani basin are tropical, with moderately cold winter and fairly hot summer. The average annual rainfall in the basin is 1305 mm, and most of the rain occurs by the influence of the southwest monsoon season i.e., between June to October. In summer, the maximum temperature goes as high as 47 °C, and in winter minimum temperature drops unto 4 °C. Brahmani River is the major source of water supply for various industries and townships and irrigation purposes in the Odisha state (India).

## Materials and methods

### Support vector machine (SVM)

**Figure 2:**
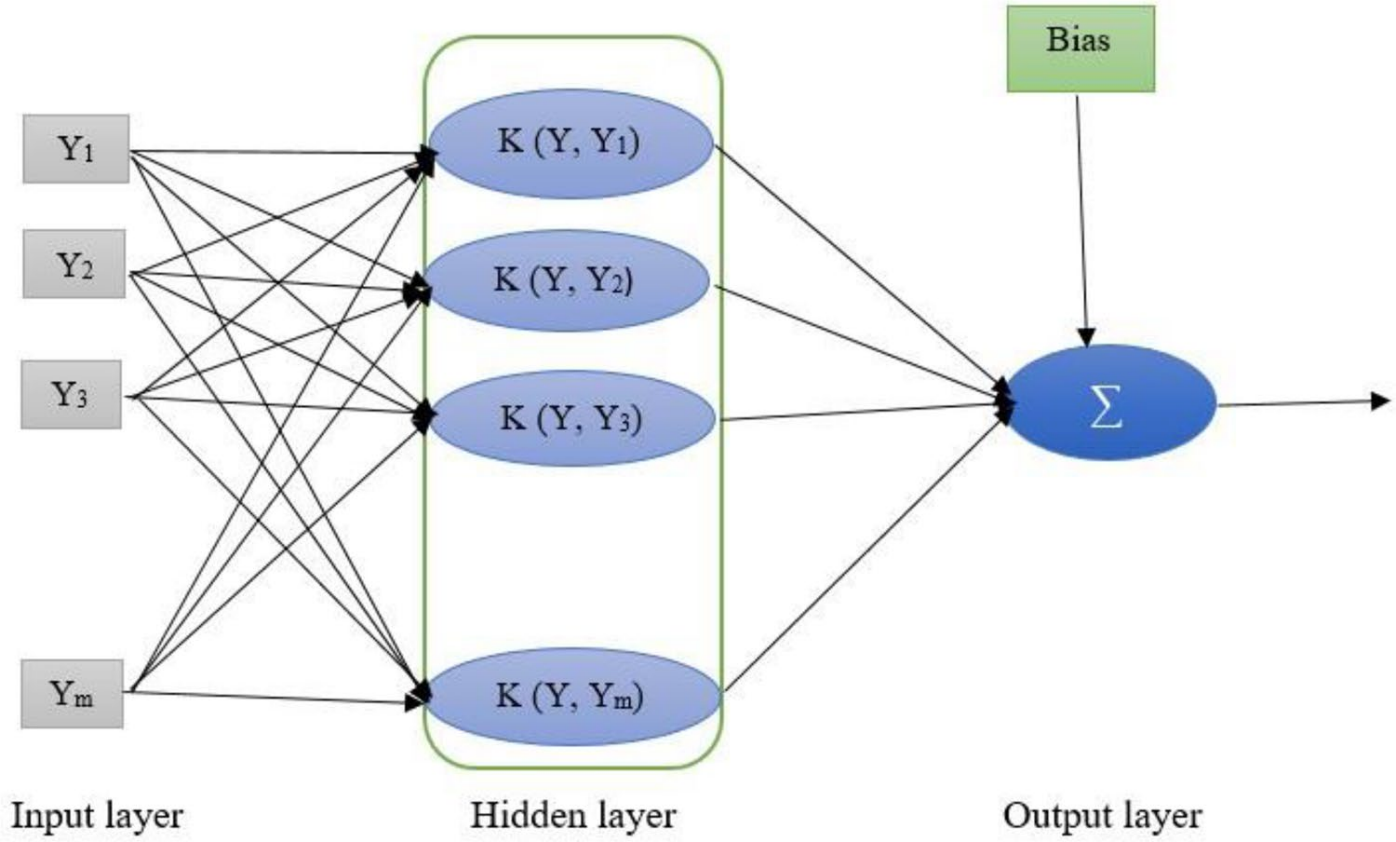
Schematic structure of SVM-based model.

SVM is a supervised binary learning algorithm first presented by Vapnik^60^. It is based on statistical learning technique and structural risk minimization. The objective for development of an SVM model is to minimize errors and model intricacy^61^. It converts input space to a high-dimensional feature space for finding the best splitting hyperplane from a training data set. The general architecture of SVM is illustrated in Fig. 2.

Amid points of two distinctive classes inside a definite error boundary, an optimum splitting hyperplane is proposed in actual space of n coordinates (${x}_{i}$ constraints in $x$ vector). Consider that $x$ and $y$ represent input and output variables. If ${x}_{i}\in {R}^{n}$, ${y}_{i}\in \{-1, 1\}$ and $i=1,\dots ,n,$ then optimum splitting hyperplane is computed utilising a categorization decision function.

$$g\left(x\right)=sgn(\sum_{i=1}^{n}{y}_{i}{\alpha }_{i}K\left({x}_{i},{x}_{j}\right)+b)$$

where $n$—number of input variables; ${\alpha }_{i}$—Lagrange multipliers; $K\left({x}_{i},{x}_{j}\right)$—kernel function; $b$—offset of hyperplane from source. Different types of kernel functions are linear, RBF, sigmoidal, or polynomial. RBF is most often used for its robust forbearance to input noise, simple design, online learning capability, and good generalisation. RBF kernel function is described using following expression^59,62^:$K\left({x}_{i},{x}_{j}\right)=\text{exp}{(-\gamma {x}_{i}-{x}_{j})}^{2}$ where $\gamma$ controls degree of nonlinearity of SVM model. Large and small $\gamma$ values cause over- and under-fitting of training data, correspondingly.

### Bat algorithm (BA)

**Figure 3:**
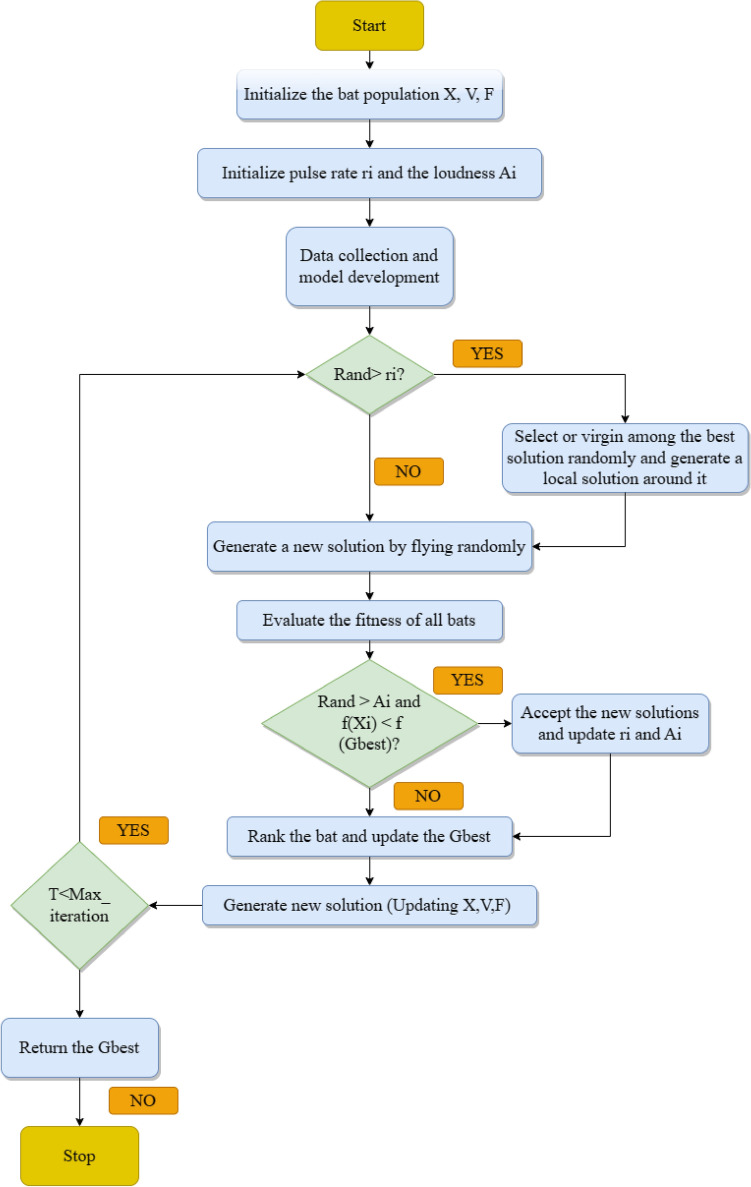
Flowchart of BA algortihm.

Yang ^63^ introduced BA emulating echolocation behaviour of a bat. In nature, there are several types of bats. When bats navigate and hunt, they all have similar behaviour; but are different in weight and size. Microbats broadly use echolocation characteristic that helps them to seek prey and avoid hurdles in complete darkness^64^. Artificial bats have a velocity vector, frequency vector, and position vector in BA, updated in the period of repetitions. BA can discover search space using velocity and position vectors (Fig. 3).

Every bat has a frequency ${F}_{i}$, velocity ${V}_{i}$, and position ${X}_{i}$ in a $d$-dimension search space. Position, frequency, and velocity vectors are updated using following equations.

1 $${V}_{i}\left(t+1\right)={V}_{i}\left(t\right)+({X}_{i}\left(t\right)-Gbest)\times {F}_{i}$$

2 $${X}_{i}\left(t+1\right)={X}_{i}\left(t\right)+{V}_{i}\left(t+1\right)$$

here $Gbest$—optimal solution obtained thus far; ${F}_{i}$–$ith$ bat’s frequency that is updated during each iteration as expressed below:

3 $${F}_{i}={F}_{min}+({F}_{max}-{F}_{min})\times \beta$$

where $\beta$—arbitrary quantity of steady distribution between 0 to 1. As given below, a random walk is employed in BA for improving its exploitation capability:

4 $${x}_{new}={x}_{old}+\varepsilon {A}_{t}$$

where $\varepsilon$—arbitrary number between − 1 to 1; $A$—intensity of produced sound. Pulse emission $(r)$ and loudness are updated at each iteration as expressed below:

5 $${A}_{i}\left(t+1\right)=\alpha {A}_{i}(t)$$

6 $${V}_{i}\left(t+1\right)={r}_{i}(0)(1-{e}^{(-\gamma \times t)})$$

where $\alpha$ and $\gamma$—constant constraints which lie amid 0 and 1 and utilised for updating pulse rate $({r}_{i})$ and loudness rate ${A}_{i}$. Pseudocode of BA is given below.

**Algorithm 1:**
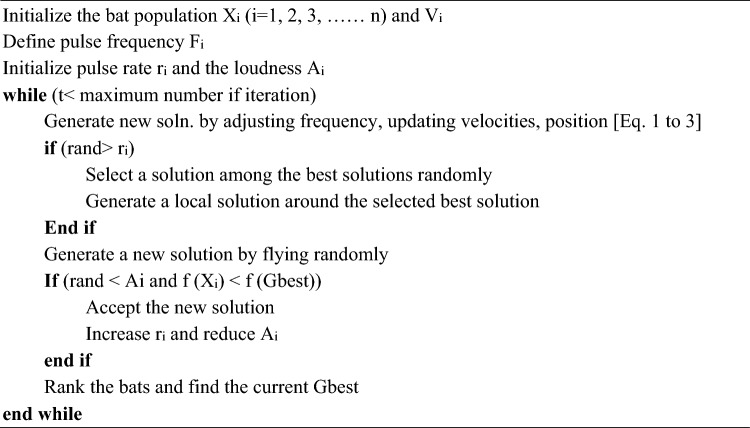
Algorithm BA

### Grasshopper optimization algorithm (GOA)

**Figure 4:**
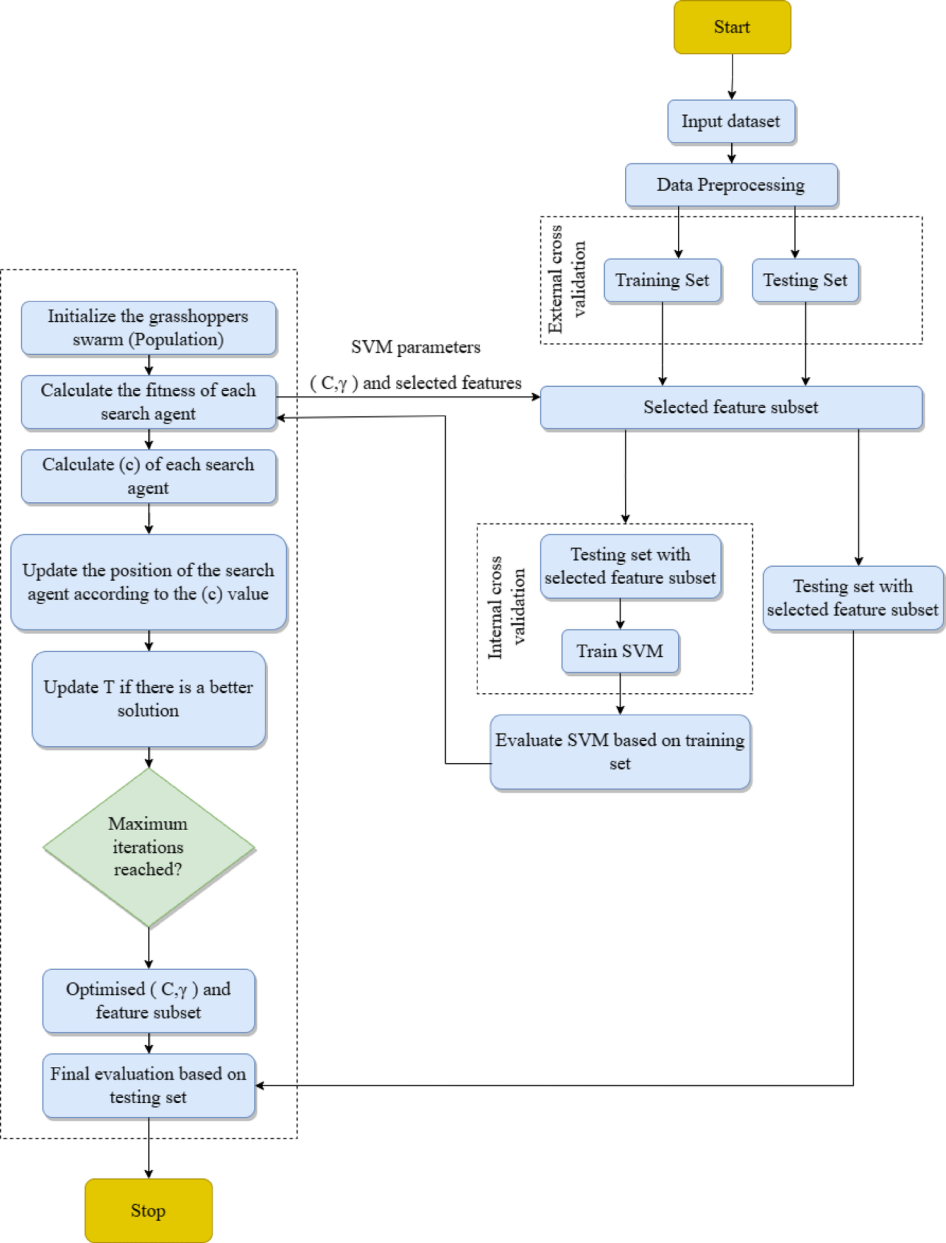
Flowchart of GOA algortihm.

Saremi et al.^65^ proposed a robust metaheuristic optimisation algorithm called the GOA mimicking the swarming behaviour of grasshoppers that comprises adults (grasshoppers having wings) and nymph (not having wings). Adults are utilised for globally searching entire search space (exploration) and finding enhanced food source areas^66^. In contrast, nymphs are utilised for exploiting a specific neighborhood or area of a specific location (exploitation). GOA efficiently balances exploitation and exploration and is mathematically incorporated in a less complicated mechanism of algorithm configuration (Fig. 4).

In nature, behaviour of grasshopper swarms that seeks food sources is articulated using following equation:

7 $${X}_{i}(t+1)=c\left[\sum_{\begin{array}{c}j=1\\ j\ne 1\\ \end{array}}^{N}c\frac{u{b}_{d}-l{b}_{d}}{2}s\left(\left|{X}_{j}(t)-{X}_{i}(t)\right|\right)\frac{{x}_{j}(t)-{x}_{i}(t)}{{d}_{ij}}\right]+\widehat{{T}_{d}}$$

where ${X}_{i}(t+1)$ —position of $i$ th grasshopper at $t + 1$ iteration; $c$—coefficient of reduction for smoothing stability amid exploitation and exploration phases. $c$ is given by:

8 $$c(t)={c}_{max}-t\frac{{c}_{max}-{c}_{min}}{{t}_{max}}$$

where ${c}_{min}$ and ${c}_{max}$—minimum and maximum values of $c(t)$ parameter, respectively. In addition, ${t}_{max}$ and $t$—maximum and current number of iterations. In Eq. (7), $l{b}_{d}$ and $u{b}_{d}$—lower and upper bound of D-dimension hunt space, —${d}_{ij}$distance between grasshoppers and $\widehat{{T}_{d}}$—location of solution having best fitness function. Lastly, $s\left(d\right)$ signifies societal forces that can be computed using:

9 $$s\left(d\right)=f{exp}^{-\frac{d}{{l}_{s}}}-\text{exp}(-d)$$

where $f$—attraction intensity and ${l}_{s}$—attractive scale of length. Additional thorough information can be found in^67,68^.

**Algorithm 2:**
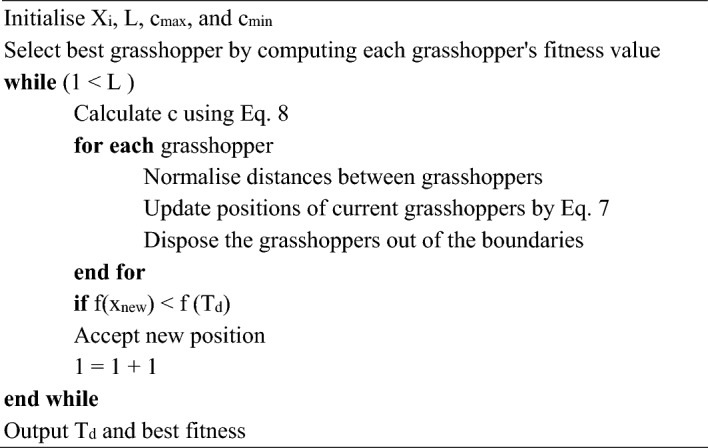
Algorithm GOA

### Butterfly optimization algorithm (*BOA*)

Arora and Singh^69^ proposed a new bionic optimization algorithm by simulating butterflies' mating and foraging behaviour, namely, BOA. The underlying operational process of BOA is based on the observation that during food search, butterflies produce specific fragrances related to their fitness. Also, the fitness of a butterfly changes accordingly as it travels from one search area to another. Fragrance is transmitted in search procedure, and meanwhile, a butterfly can recognize variations in the fragrance of other butterflies^70^. Butterflies travel in the direction of the one butterfly with a more potent fragrance in global search. At the same time, butterflies move arbitrarily in local search for searching food when they cannot sense fragrance from other butterflies. Mainly, fragrance having an exclusive aroma in every butterfly is the distinctive feature of BOA that can be expressed as in Eq. (10):

10 $$f={cl}^{a}$$

where $f$—detected fragrance magnitude, that is, how other butterflies detect strong fragrances; $c$—sensory modality; $l$—intensity of stimulus; $a$—power proponent which depends on modality accounting changing grade of absorption. In BOA, there are two key steps: global and local search phases. Butterfly takes a step in the direction of fittest solution/butterfly ${g}^{*}$ in global search and is expressed utilising Eq. (2).

11 $${x}_{i}^{t+1}={x}_{i}^{t}+({r}^{2}\times {g}^{*}-{x}_{i}^{t})\times {f}_{i}$$

where ${x}_{i}^{t}$—solution vector ${x}_{i}$ in iteration $t$ for $ith$ butterfly; $g*$—current optimal solution obtained between all solutions in present iteration.$r$—arbitrary number between 0 and 1; ${f}_{i}$—fragrance of $ith$ butterfly.

**Figure 5:**
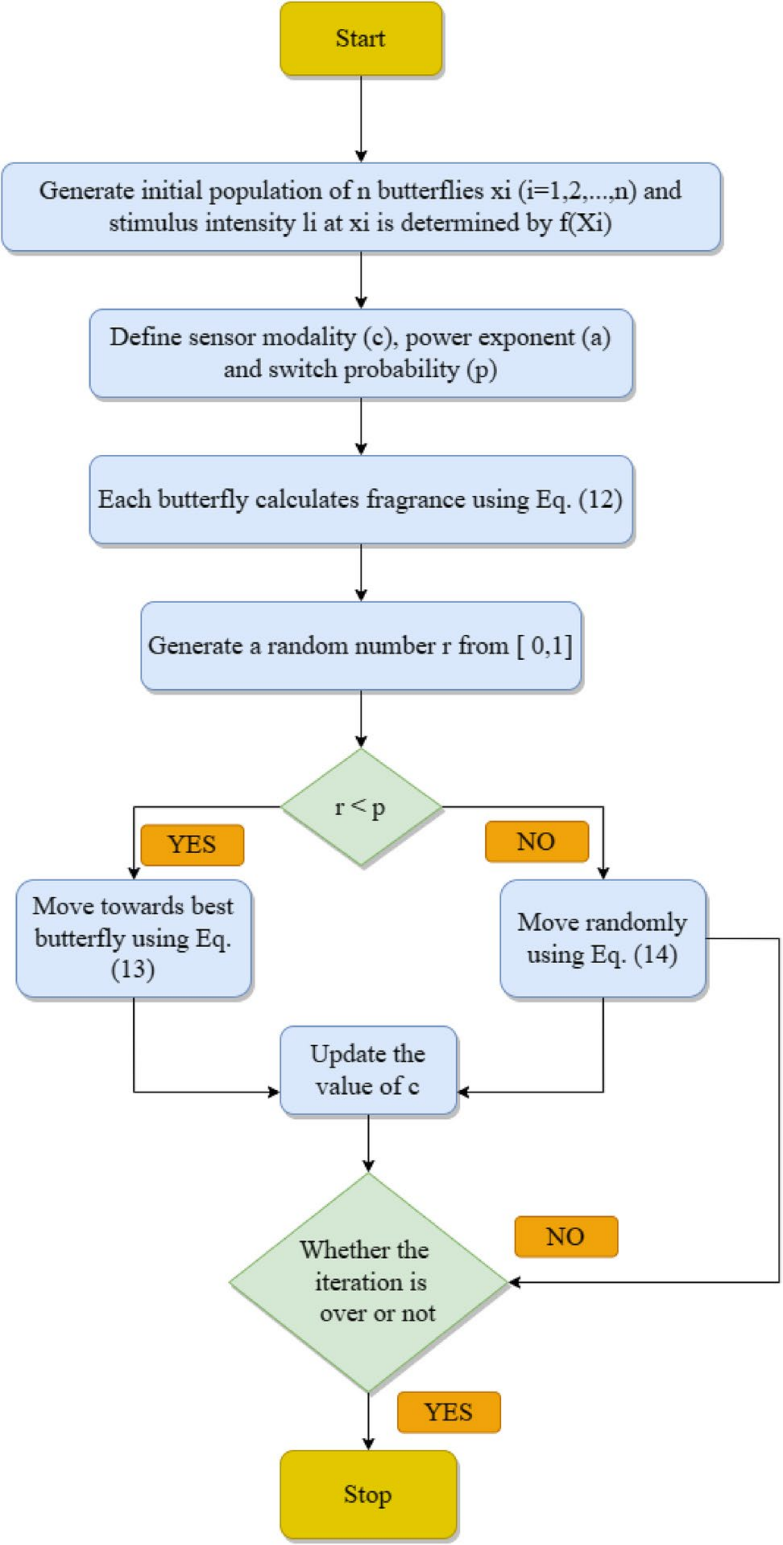
Flowchart of BOA algortihm.

Local search is formulated using following equation:

12 $${x}_{i}^{t+1}={x}_{i}^{t}+({r}^{2}\times {x}_{j}^{t}-{x}_{k}^{t})\times {f}_{i}$$

where ${x}_{j}^{t}$ and ${x}_{k}^{t}$—$jth$ and $kth$ butterflies from solution space. Figure 5 provides a basic flow diagram of the algorithm.

**Algorithm 3:**
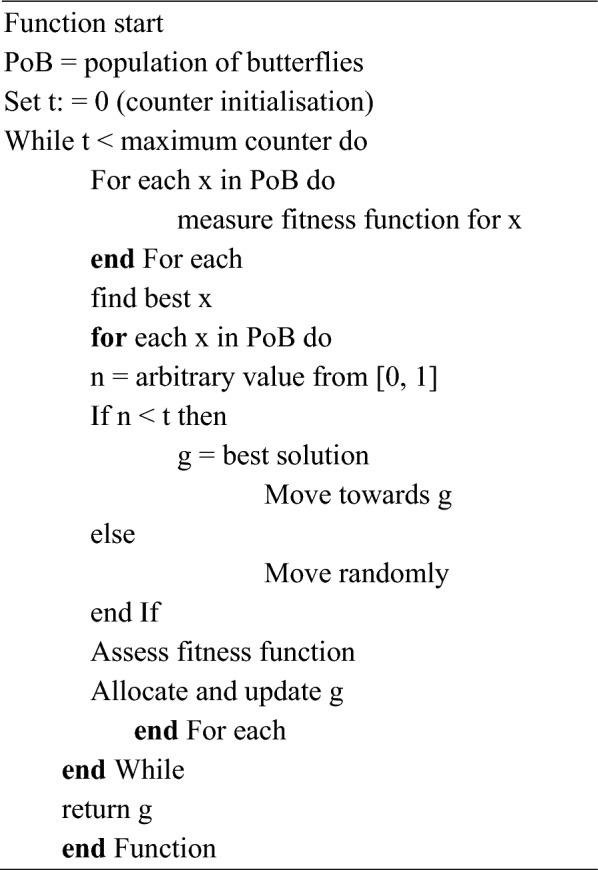
Algorithm BOA

### Sparrow search algorithm (SSA)

Xue and Shen^71^ proposed SSA based on theory of anti-predation and foraging behavior of sparrows. SSA is novel and has advantages like fast convergence speed and strong optimisation capability. Mainly the procedure of sparrow foraging is simulated by SSA. The procedure is a producer-joiner model, and it overlays the early warning and detection mechanism^72^. Producers are those individual sparrows who find food without difficulty, and other entities are joiners. A specific number of sparrows in the population are chosen for early warning and investigation at the same time. However, food is abandoned if danger is found since safety is the priority.

Mathematical Model of Sparrow Search Algorithm.

Individuals can be categorized as alerters, participants, or discoverers in SSA. The discoverer is in charge of organizing the population's hunt and locating food. In order to grab food, the participants follow the discoverer. When environmental dangers arise, the alerter notifies the sparrow population to flee to a safe location.

It is necessary to create the following rules to simplify the behavior of the sparrow in order to represent the eating process of the bird using a mathematical model:

i. The objective function's fitness evaluation determines the environment's fitness in the sparrow population, and the finder's fitness is greater than the participants'.
ii. The discoverer and the participant have an internal competitive relationship. In an attempt to boost their own energy, some participants watch how the discoverer behaves in order to compete for food.
iii. Less energetic sparrows may relocate in search of more energetic ones.
iv. Sparrows possess adaptable individual behavioral methods that enable them to alternate between participants and discoverers, rendering them highly fit discoverers; yet, the population's proportion of participants and discoverers does not change.
v. When a sparrow population's alarm value exceeds the security threshold, the finder flees from its current location and guides the population to a secure spot. Warners in the population warn when they perceive an external environmental threat.
vi. In order to minimize the risk of their own predation, the alert will take the initiative in escaping when it detects external environmental threats or natural enemies. The alert at the population center will randomly transition from a feeding state to an active state, while the alert at the population edge will move closer to the population center.

**Step 1:** Construct and set up the solution. At this point, it is known the size of the population, the maximum number of replicates, the producer ratio (PD), and the PV (sparrows in intensive care) ratio. Equation (13) displays the sparrow population's starting position. They are generated at random.

13 $$X=\left[\begin{array}{ccccc}{x}_{\text{1,1}}& {x}_{\text{1,2}}& \cdots & \cdots & {x}_{1,d}\\ {x}_{\text{2,1}}& {x}_{\text{2,2}}& \cdots & \cdots & {x}_{2,d}\\ \vdots & \vdots & \vdots & \vdots & \vdots \\ {x}_{n,1}& {x}_{n,2}& \cdots & \cdots & {x}_{n,d}\end{array}\right]$$

In Eq. (13), $n$—number of sparrows; $d$—dimension of choice variables. Equation (14) is used to assess each person's suitability for the upcoming operation. Each row in ${F}_{X}$ represents a person's fit, and $n$ in Eq. (14) indicates number of sparrows:

14 $${F}_{X}=\left[\begin{array}{ccccc}{f[x}_{\text{1,1}}& {x}_{\text{1,2}}& \cdots & \cdots & {x}_{1,d}]\\ f[{x}_{\text{2,1}}& {x}_{\text{2,2}}& \cdots & \cdots & {x}_{2,d}]\\ \vdots & \vdots & \vdots & \vdots & \vdots \\ {f[x}_{n,1}& {x}_{n,2}& \cdots & \cdots & {x}_{n,d}]\end{array}\right]$$

**Step 2:** Those who create cuisine are not given favor over producers with greater fitness values in the SSA. Unlike the explorers, producers are able to seek a wider area for cuisine because they are in charge of locating it and guiding the movement of the entire population. In SSA, the discoverer’s location update formula is expressed using

15 $${X}_{i}^{t+1}=\left\{\begin{array}{c}{X}_{i}^{t}\text{exp}\left(-\frac{i}{a{t}_{max}}\right), ifR<S\\ {X}_{i, j}+QL, if R\ge S\end{array}\right.$$

where $t$—current iteration number, and ${X}_{i}$—information about $i$ th sparrow’s position. $a$—arbitrary number between [0, 1].$S(S\in [0.5, 1])$ and $R(R\in [0, 1])$ signify safety and warning parameters, correspondingly. $R$—arbitrary number, $S$—specified constant. When $R<S$, search environment is found to be safe, and no danger for the population, and a broad range of searches can be conducted by discoverer. When $R \ge S$, adjust search approach as scouts find a threat and hence, rapidly move closer to an innocuous region. $Q$—an arbitrary number following a normal distribution. $L$—an all-one matrix of $1 \times d$ dimension.

Joiner’s position update formula is formulated by

16 $${X}_{i}^{t+1}=\left\{\begin{array}{c}Qexp\left(\frac{{X}_{r}-{X}_{i}^{t}}{{i}^{2}}\right),if i>\frac{n}{2}\\ {X}_{b}^{t+1}+\left|{X}_{i}^{t}-{X}_{b}^{t+1}\right|{A}^{+}L, otherwise\end{array}\right.$$

where ${X}_{b}$—best location of producer at present and ${X}_{r}$—worst location in the world at present. $A$ -matrix of $1 \times d$ dimensions, every element has 1 or − 1 value and ${A}^{+}={A}^{T}{(A{A}^{T})}^{-1}$.

**Figure 6:**
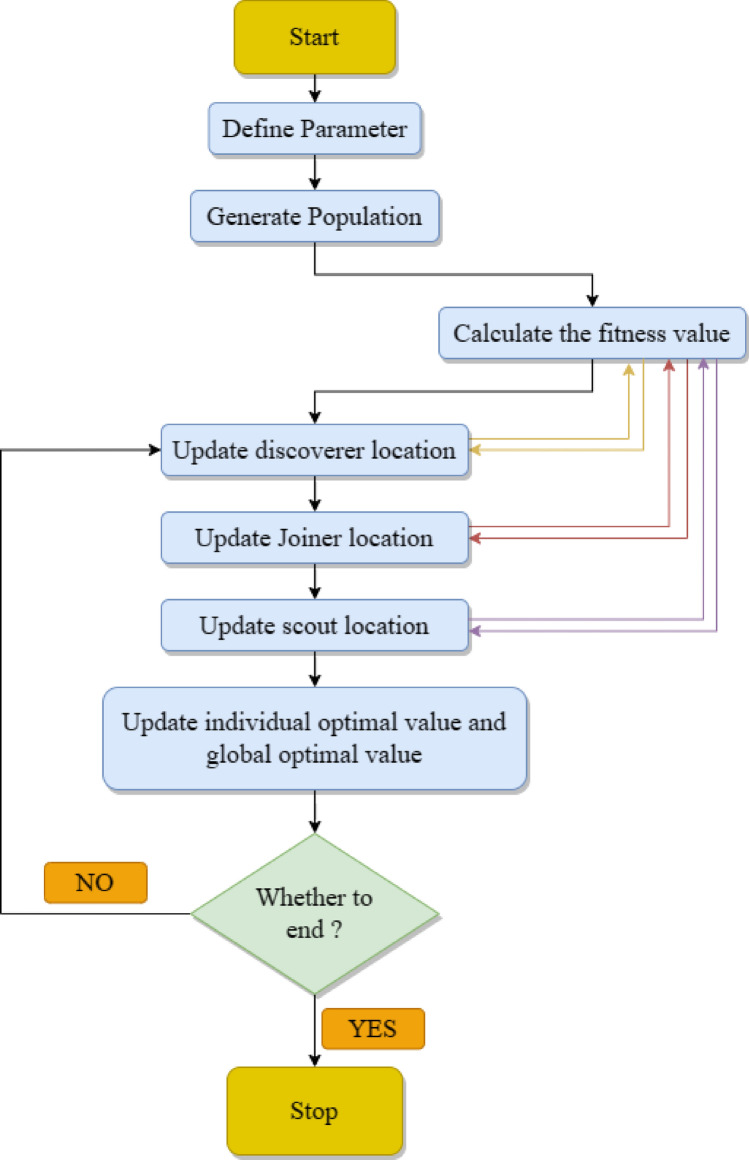
Flowchart of SSA algortihm.

Scout’s position update formula formulated by

17 $${X}_{i}^{t+1}=\left\{\begin{array}{c}{X}_{B}^{t}+\beta \left|{X}_{i}^{t}-{X}_{B}^{t}\right|, if {f}_{i}\ne {f}_{B}\\ {X}_{i}^{t}+K\frac{\left|{X}_{i}^{t}-{X}_{r}^{t}\right|}{\left({f}_{i}-{f}_{R}\right)+\varepsilon }, if {f}_{i}={f}_{B}\end{array}\right.$$

where ${X}_{B}$—current global optimal position; $\beta$—steplength regulator parameter, an arbitrary number with variance ‘1’ and mean value ‘0’ drawn on a normal distribution. $K$—arbitrary number between [− 1, 1]. ${f}_{i}$—distinct fitness value of sparrow at present step. ${f}_{R}$ and ${f}_{B}$—current worst fitness and global optimal values, correspondingly. $\varepsilon$—a tiny constant. At the end of the iteration, optimisation result is output. Flowchart of SSA is given in Fig. 6.

**Algorithm 4:**
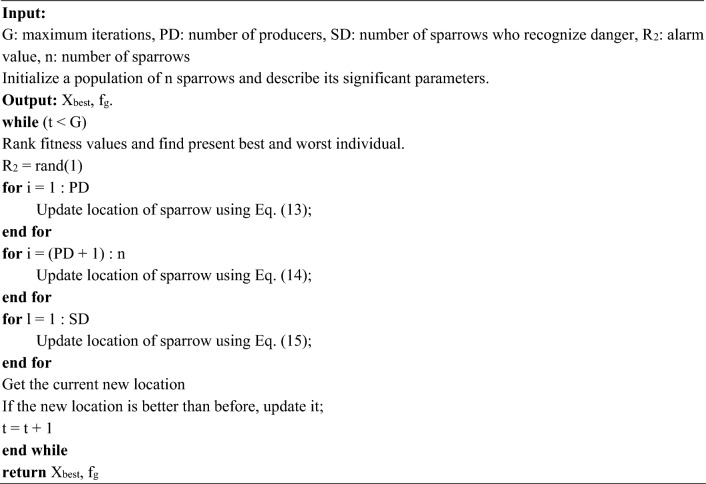
Algorithm SSA

During iteration procedure, if new position of sparrow is improved than preceding position, present position will be updated and global optimal fitness and global optimal position are found. Also, the sparrow’s identity is continuously updated and alternated during this phase. If each sparrow is well adapted, it can be a finder; however, proportions of joiners and finders in the population are constant.

### Performance criteria

Four performance indices that includes R^2^, RMSE, MAE, and E_NS_ are considered in this study for measuring the accuracy of the applied models ^73,74^. The mathematical expression of the statistical measures can be denoted as:

18 $${\text{R}}^{2}={(\frac{\sum_{i=1}^{N}({O}_{i} -\overline{{O }_{i}})({F}_{i} -\overline{{F }_{i}})}{\sqrt{\sum_{i=1}^{N}{({O}_{i} -\overline{{O }_{i}})}^{2}\sum_{i=1}^{N}{({F}_{i} -\overline{{F }_{i}})}^{2}}})}^{2}$$

19 $$\text{MAE }=\frac{1}{N}\sum_{i=1}^{N}\left|{F}_{i}-{O}_{i}\right|$$

20 $$\text{RMSE }=\sqrt{\frac{1}{n}\sum_{i=1}^{n}{\left[{O}_{i}-{F}_{i}\right]}^{2}}$$

21 $$\text{ENS }=1-[\frac{\sum_{\text{i}=1}^{\text{N}}{\left({O}_{i}-{F}_{i}\right)}^{2}}{{\sum }_{\text{i}=1}^{\text{N}}{(\left|{O}_{i} -\overline{{O }_{i}}\right|)}^{2}}]$$

where ${O}_{i}$; ${F}_{i}$; $\overline{{O }_{i}}$ and $\overline{{F }_{i}}$ express observed, predicted, average observed and average predicted values.

R^2^ is a statistical index in regression, which shows how fittingly the predictive models estimate real data sets. When the value of R^2^ is 1, the predicted values perfectly fit observed values, whereas value of 0 specifies no linear connection. The average error magnitude is measured by a quadratic scoring rule known as the RMSE. It is the square root of average of squared difference amid predicted and actual observations. Contrary to RMSE, MAE is a quantity utilized for measuring how closer the prediction values are to actual observations. MAE calculates average error magnitude amid prediction and observed values with no difference amid direction of error. Low values of MAE and RMSE specify high assurance in model-prediction values^69^. RMSE has the advantage of penalizing huge errors more, thus can be extra suitable in certain circumstances. On the other hand, MAE is evidently a better statistical measure from an interpretation viewpoint. In addition, E_NS_ is one of the standardized measures to assess the model precision, whose value lies between zero and one. E_NS_ value of 1 specifies best agreement, while a value of 0 specifies no agreement. E_NS_ measure is extremely subtle to limit values because of usage of difference squares.

For each model scenario, Table 1 gives input parameter combinations where discharge and suspended sediment load parameters are given in current time step (Q_t_ and SSL_t_) along with previous monthly lag time. From Table 1 it can be seen that five different scenarios were considered for estimating SSL utilising different input combinations of SSL_t−1_, SSL_t−2_, SSL_t−3_, Q_t_, Q_t−1_, Q_t−2_, Q_t−3_ parameters. It is worthy to note that the selected scenarios are considered based on correlation of Q and SSL variables.

**Table 1:** Implemented models and their input parameters.

Input parameters	SVM-based models
SSL_t_, Q_t_	SVM1, SVM-BA1, SVM-GOA1, SVM-BOA1, SVM-SSA1
SSL_t−1_, Q_t−1_	SVM2, SVM-BA2, SVM-GOA2, SVM-BOA2, SVM-SSA2
SSL_t−1_, SSL_t−2_, Q_t−1_, Q_t−2_	SVM3, SVM-BA3, SVM-GOA3, SVM-BOA3, SVM-SSA3
SSL_t−1_, SSL_t−2_, SSL_t−3_, Q_t−1_, Q_t−2_, Q_t−3_	SVM4, SVM-BA4, SVM-GOA4, SVM-BOA4, SVM-SSA4
SSL_t−1_, SSL_t−2_, SSL_t−3_, Q_t_, Q_t−1_, Q_t−2_, Q_t−3_	SVM5, SVM-BA5, SVM-GOA5, SVM-BOA5, SVM-SSA5

## Modeling results and analysis

Four hybrid SVM models employed in this study in integration with four different MAs namely BA, BOA, GOA, and SSA are compared for modeling SSL utilizing data of River Brahmani basin. Next, performance of the applied models was assessed against each other and conventional SVM model using statistical measures and graphical interpretations. This section provides the outcomes of the comparisons and effectiveness of all mentioned models at four proposed gauge stations. Two major influencing data, i.e., Q, and SSL, were applied to predict SSL. The outcomes of training and testing periods are provided in Tables 2, 3, 4, 5 and 6, showing prediction performance of all applied models on basis of R^2^, RMSE, E_NS_, and MAE criteria. The results obtained indicated that these proposed parameters effectively estimated the SSL.

**Table 2:** Performance of SVM models for SSL estimation for all data.

Station	Input	R^2^		E_NS_		MAE		RMSE	
		Training	Testing	Training	Testing	Training	Testing	Training	Testing
Tilga	SVM1	0.9114	0.8993	0.906	0.8942	35.2145	40.8523	35.3499	40.9874
	SVM2	0.9151	0.9029	0.9106	0.8986	34.4698	39.6318	34.6043	39.7661
	SVM3	0.9179	0.9054	0.9132	0.9012	33.217	38.6214	33.3517	38.7556
	SVM4	0.9191	0.9065	0.914	0.90155	32.368	37.5632	32.5031	37.6991
	SVM5	0.9198	0.9076	0.9155	0.902	31.55	36.3214	31.6843	36.457
Jenapur	SVM1	0.9111	0.8991	0.9071	0.8942	35.6214	40.9317	35.7554	41.0666
	SVM2	0.9147	0.9027	0.9094	0.8992	34.6317	39.9317	34.767	40.0652
	SVM3	0.91582	0.9038	0.91242	0.8976	33.5319	38.9321	33.6653	39.0683
	SVM4	0.9188	0.9063	0.9139	0.9	32.4952	37.8167	32.6301	37.953
	SVM5	0.9196	0.9074	0.9161	0.903	31.9641	36.5631	32.0976	36.6975
Jaraikela	SVM1	0.9116	0.8996	0.9068	0.8941	34.9005	40.6325	35.0353	40.768
	SVM2	0.9155	0.9032	0.9095	0.8975	34.218	39.341	34.354	39.4767
	SVM3	0.9183	0.9056	0.9125	0.9018	32.8414	38.3517	32.9772	38.4855
	SVM4	0.9193	0.9069	0.9138	0.903	32.216	37.218	32.3515	37.3519
	SVM5	0.92014	0.90819	0.91444	0.90449	31.4562	35.9001	31.5919	36.0338
Gomlai	SVM1	0.9107	0.8987	0.9042	0.8946	35.892	41.13	36.0285	41.2641
	SVM2	0.91195	0.90012	0.90535	0.89402	34.819	40.53118	34.9556	40.66728
	SVM3	0.9157	0.9035	0.9105	0.8999	33.8415	39.0017	33.9767	39.1353
	SVM4	0.9185	0.9059	0.9144	0.9013	32.7558	38.1106	32.7558	38.2452
	SVM5	0.9194	0.907	0.9133	0.9007	32.0014	36.964	32.1375	37.1004

**Table 3:** Performance of SVM-BA models for SSL estimation for all data.

Station	Input	R^2^		E_NS_		MAE		RMSE	
		Training	Testing	Training	Testing	Training	Testing	Training	Testing
Tilga	SVM-BA-1	0.97104	0.9321	0.96534	0.9279	14.7633	31.004	14.899	31.1382
	SVM-BA-2	0.9719	0.9341	0.9681	0.9282	13.9362	30.2037	14.07	30.3396
	SVM-BA-3	0.9747	0.9375	0.9708	0.9319	13.3517	29.5139	13.4856	29.6495
	SVM-BA-4	0.97586	0.93895	0.97216	0.93355	12.6325	28.7021	12.7662	28.8375
	SVM-BA-5	0.9778	0.9425	0.974	0.9381	12.03497	27.963	12.16877	28.0975
Jenapur	SVM-BA-1	0.9708	0.9319	0.9665	0.9257	14.9049	31.2205	15.0392	31.3567
	SVM-BA-2	0.9718	0.9339	0.9676	0.9276	14.197	30.6685	14.3312	30.8048
	SVM-BA-3	0.97264	0.93481	0.96674	0.93041	13.49	29.6312	13.6259	29.7656
	SVM-BA-4	0.9754	0.9385	0.9698	0.9345	12.8001	28.9214	12.9357	29.0554
	SVM-BA-5	0.9776	0.9423	0.9722	0.9369	12.164	28.1134	12.2994	28.2487
Jaraikela	SVM-BA-1	0.9713	0.9324	0.9677	0.9286	14.5001	30.951	14.6337	31.0848
	SVM-BA-2	0.9721	0.9344	0.9675	0.9305	13.8395	30.194	13.9741	30.3279
	SVM-BA-3	0.9749	0.9379	0.9685	0.9342	13.1138	29.3148	13.2502	29.4485
	SVM-BA-4	0.9769	0.9418	0.9704	0.937	12.53268	28.5319	12.66918	28.4583
	SVM-BA-5	0.97817	0.9431	0.97357	0.93782	11.8112	27.812	11.9458	27.948
Gomlai	SVM-BA-1	0.9705	0.9315	0.967	0.9279	15.11734	31.36	15.25084	31.4936
	SVM-BA-2	0.97165	0.93252	0.96636	0.92792	14.36948	30.79	14.50568	30.9246
	SVM-BA-3	0.9725	0.9345	0.9662	0.9281	13.6874	29.9416	13.8237	30.078
	SVM-BA-4	0.9751	0.9382	0.9707	0.9317	12.9103	29.147	13.0447	29.2835
	SVM-BA-5	0.9772	0.9422	0.9732	0.9355	12.36974	28.3217	12.50374	28.6667

**Table 4:** Performance of SVM-GOA models for SSL estimation for all data.

Station	Input	R^2^		E_NS_		MAE		RMSE	
		Training	Testing	Training	Testing	Training	Testing	Training	Testing
Tilga	SVM-GOA-1	0.9789	0.9439	0.975	0.9392	11.311	26.8019	11.4449	26.9366
	SVM-GOA-2	0.9795	0.9451	0.9758	0.9414	10.737	25.9317	10.8707	26.0654
	SVM-GOA-3	0.9807	0.9481	0.9759	0.9433	10.1364	25.1348	10.2712	25.2696
	SVM-GOA-4	0.98239	0.9492	0.97829	0.94466	9.5368	24.3645	9.6709	24.5005
	SVM-GOA-5	0.983	0.9507	0.9769	0.9448	8.93214	23.5147	9.06824	23.6505
Jenapur	SVM-GOA-1	0.9788	0.9435	0.9729	0.9401	11.53985	27.3216	11.5398	27.455
	SVM-GOA-2	0.97921	0.9447	0.97361	0.9391	10.83	26.1395	10.9656	26.2751
	SVM-GOA-3	0.98029	0.94604	0.97489	0.94064	10.27	25.3268	10.4054	25.4622
	SVM-GOA-4	0.9821	0.9489	0.9763	0.9444	9.7009	24.5214	9.8367	24.6559
	SVM-GOA-5	0.9827	0.9503	0.9772	0.9456	9.0013	23.8541	9.1368	23.9888
Jaraikela	SVM-GOA-1	0.97913	0.9441	0.97273	0.9383	11.26649	26.6632	11.4028	26.799
	SVM-GOA-2	0.9798	0.9454	0.9733	0.9389	10.53176	25.7062	10.6682	25.8427
	SVM-GOA-3	0.9811	0.9483	0.9777	0.9417	9.92	24.93214	10.0534	25.0687
	SVM-GOA-4	0.98254	0.9496	0.97764	0.9444	9.4937	24.2103	9.6286	24.3455
	SVM-GOA-5	0.98312	0.9508	0.97962	0.94662	8.84129	23.26	8.97479	23.3941
Gomlai	SVM-GOA-1	0.9783	0.9433	0.972	0.9381	11.6206	27.5001	11.7569	27.6353
	SVM-GOA-2	0.97914	0.94423	0.97474	0.93983	10.9004	26.34804	11.0348	26.4824
	SVM-GOA-3	0.97996	0.9458	0.97596	0.9418	10.43198	25.5109	10.5659	25.6449
	SVM-GOA-4	0.98156	0.9485	0.97686	0.9432	9.81473	24.785	9.94943	24.9203
	SVM-GOA-5	0.9826	0.9501	0.9775	0.9467	9.281	23.9934	9.4161	24.1268

**Table 5:** Performance of SVM-BOA models for SSL estimation for all data.

Station	Input	R^2^		E_NS_		MAE		RMSE	
		Training	Testing	Training	Testing	Training	Testing	Training	Testing
Tilga	SVM-BOA-1	0.9847	0.9516	0.9811	0.9461	8.295	22.5147	8.4286	22.6502
	SVM-BOA-2	0.9854	0.9538	0.9817	0.9481	7.6325	21.6301	7.7662	21.7658
	SVM-BOA-3	0.9862	0.9546	0.9814	0.949	6.681	20.9327	6.8158	21.0683
	SVM-BOA-4	0.98715	0.95563	0.98115	0.95023	5.3961	20.36	5.5321	20.4954
	SVM-BOA-5	0.9897	0.9593	0.9831	0.9547	4.449	19.53218	4.5856	19.66668
Jenapur	SVM-BOA-1	0.9844	0.9514	0.9787	0.9463	8.46325	22.9641	8.59895	23.0992
	SVM-BOA-2	0.9852	0.9534	0.9796	0.9491	7.7398	21.8124	7.8754	21.9467
	SVM-BOA-3	0.9859	0.95434	0.9801	0.94994	6.8315	21.22108	6.9669	21.35548
	SVM-BOA-4	0.9869	0.9554	0.9824	0.9514	5.7934	20.5183	5.9279	20.6523
	SVM-BOA-5	0.9895	0.9588	0.9847	0.9536	4.79214	19.9854	4.92694	20.1207
Jaraikela	SVM-BOA-1	0.9848	0.9519	0.9786	0.9458	8.1367	22.3218	8.2729	22.4579
	SVM-BOA-2	0.9855	0.9541	0.979	0.9505	7.53321	21.5987	7.66971	21.7323
	SVM-BOA-3	0.9864	0.9549	0.9798	0.9512	6.32567	20.849	6.46227	20.9827
	SVM-BOA-4	0.9886	0.9583	0.9834	0.9535	5.1377	20.2945	5.2729	20.4293
	SVM-BOA-5	0.99019	0.9596	0.98489	0.95341	4.2796	19.3658	4.4149	19.5018
Gomlai	SVM-BOA-1	0.9839	0.9511	0.9796	0.9462	8.66	23.1347	8.7943	23.2696
	SVM-BOA-2	0.9851	0.95211	0.9807	0.94861	7.93148	22.0364	8.06588	22.1699
	SVM-BOA-3	0.9857	0.9542	0.9817	0.948	7.3218	21.352	7.4558	21.4882
	SVM-BOA-4	0.9866	0.9551	0.9813	0.9486	5.93476	20.632	6.07006	20.7685
	SVM-BOA-5	0.9891	0.9584	0.9857	0.9519	4.93	20.0019	5.0634	20.1385

**Table 6:** Performance of SVM-SSA models for SSL estimation for all data.

Station	Input	R^2^		E_NS_		MAE		RMSE	
		Training	Testing	Training	Testing	Training	Testing	Training	Testing
Tilga	SVM-SSA-1	0.9911	0.9634	0.9859	0.9587	3.1059	18.5841	3.2411	18.7188
	SVM-SSA-2	0.99203	0.9645	0.98793	0.9594	2.0014	17.8213	2.1355	17.8213
	SVM-SSA-3	0.9928	0.9675	0.9867	0.9632	0.7965	16.93247	0.9326	17.06677
	SVM-SSA-4	0.99401	0.9685	0.99051	0.96472	0.22367	16.28946	0.35717	16.42526
	SVM-SSA-5	0.9958	0.9699	0.9896	0.9643	0.03648	15.5637	0.17268	15.6992
Jenapur	SVM-SSA-1	0.9908	0.9631	0.9848	0.9597	3.4698	18.9354	3.6058	19.0688
	SVM-SSA-2	0.99184	0.9644	0.98604	0.9595	2.35698	18.0062	2.49278	18.1411
	SVM-SSA-3	0.99258	0.96529	0.98708	0.96179	0.9631	17.1139	1.0986	17.2474
	SVM-SSA-4	0.9937	0.9683	0.988	0.9621	0.35698	16.365	0.49268	16.5012
	SVM-SSA-5	0.9954	0.9694	0.9918	0.9643	0.07965	15.7792	0.21325	15.9143
Jaraikela	SVM-SSA-1	0.9914	0.9636	0.988	0.9578	2.8463	18.36	2.9797	18.4958
	SVM-SSA-2	0.9921	0.9648	0.9872	0.9593	1.86321	17.5596	1.1998	17.6951
	SVM-SSA-3	0.9931	0.9679	0.9896	0.9622	0.521	16.7708	0.6545	16.9065
	SVM-SSA-4	0.9946	0.9691	0.9903	0.965	0.196	16.0031	0.3303	16.1372
	SVM-SSA-5	0.99616	0.97014	0.99195	0.96481	0.01578	15.3926	0.14994	15.5287
Gomlai	SVM-SSA-1	0.9905	0.9629	0.986	0.9577	3.88432	19.2147	4.01882	19.3499
	SVM-SSA-2	0.9916	0.96418	0.9869	0.96008	2.562	18.2249	2.6967	18.359
	SVM-SSA-3	0.9924	0.96502	0.9873	0.95892	1.0647	17.3524	0.9685	17.4885
	SVM-SSA-4	0.9935	0.9681	0.9892	0.9645	0.4935	16.59743	0.6278	16.73103
	SVM-SSA-5	0.9951	0.9693	0.9894	0.9644	0.12984	15.88395	0.26554	16.01885

The performance statistics of SVM1 during testing phase for Jaraikela station when SSL and discharge of current month is considered are as follows: R^2^ = 0.8996, E_NS_ = 0.8941, MAE = 40.6325, RMSE = 40.768, respectively. Next, we consider a combination of SSL_t−1_, Q_t−1_ (one month lag). Here the SVM2 model (R^2^ = 0.9.32, E_NS_ = 0.8975, MAE = 39.341, RMSE = 39.4767) performs better than the SVM1 model. Similarly, when a combination of SSL_t−1_, SSL_t−2_, SSL_t−3_, Q_t_, Q_t−1_, Q_t−2_, Q_t−3_ (one-month, two-month, three-month lag and current month) are considered, the performance of this combination are: R^2^ = 0.90819, E_NS_ = 0.90449, MAE = 35.9001, RMSE = 36.0338. It can be observed that, as the lag time is increased, there is a gradual performance improvement in case of SVM as shown in Table 2, i.e., the prediction accuracy of SVM5 model is better than the prediction accuracy of SVM4, SVM3, SVM2 and which in turn is better than SVM1 model.

The performance statistics of SVM-BA1, SVM-GOA1, SVM-BOA1 when SSL and discharge of current month is considered are as follows: R^2^ = 0.9324, E_NS_ = 0.9286, MAE = 30.951, RMSE = 31.0848; R^2^ = 0.9441, E_NS_ = 0.9383, MAE = 26.6632, RMSE = 26.799; R^2^ = 0.9514, E_NS_ = 0.9458, MAE = 22.3218, RMSE = 22.4579 for Jaraikela station during the testing phase. When a combination of SSL_t−1_, Q_t−1_ is considered, the SVM-BA2, SVM-GOA2, SVM-BOA2 generates R^2^ = 0.9344, E_NS_ = 0.9305, MAE = 30.194, RMSE = 30.3279; R^2^ = 0.9454, E_NS_ = 0.9389, MAE = 25.7062, RMSE = 25.8427; R^2^ = 0.9541, E_NS_ = 0.9505, MAE = 21.5987, RMSE = 21.7323 as compared to first model scenario. Further, the SVM-BA5, SVM-GOA5, SVM-BOA5 models gives R^2^ (0.9341), E_NS_ (0.93782), MAE (27.812), RMSE (27.948); R^2^ (0.9508), E_NS_ (0.94662), MAE (23.26), RMSE (23.3941); R^2^ (0.9596), E_NS_ (0.95341), MAE (19.3658), RMSE (19.5018) when we consider a combination of SSL_t−1_, SSL_t−2_, SSL_t−3_, Q_t_, Q_t−1_, Q_t−2_, Q_t−3_. Thus in case of SVM-BA, SVM-GOA, SVM-BOA too, the last scenario performs better than the other four scenarios. The detailed results are shown in Tables 3, 4 and 5.

By observing Table 6, considering SSL_t−1_, SSL_t−2_, SSL_t−3_, Q_t_, Q_t−1_, Q_t−2_, Q_t−3_ provides best results i.e., R^2^ = 0.97014, E_NS_ = 0.96481, MAE = 15.3926, RMSE = 15.5287 followed by SSL_t−1_, SSL_t−3_, SSL_t−2_, SSL_t−3_, Q_t−3_ (R^2^ = 0.9691, E_NS_ = 965, MAE = 16.0031, RMSE = 16.1372), and the worst performance was obsered when SSL_t_, Q_t_ is considered (R^2^ = 0.9636, E_NS_ = 0.9578, MAE = 18.36, RMSE = 18.4958). The performance statistics of SVM-SSA method is best compared to all other hybrid models for all the stations during both training and testing phases. From all the selected stations, all the models perfomed best at Jaraikela, Tilga, Jenapur and Gomlai respectively.

Tables 2, 3, 4, 5 and 6 provide the outcomes on train and test datasets for the applied techniques. It gives a general trend, where performance (R^2^, E_NS_, MAE, RMSE) tends to rise when more characteristics are included, which is found for all proposed models at all the selected stations utilized for SSL prediction. Based on type of statistical measures, the larger values of E_NS_ or R^2^ signify that results are better whereas smaller values of RMSE or MAE signify that obtained results are better. Tables 2, 3, 4, 5 and 6 provide the outcomes of statistical assessment measures for result data of Tilga, Jenapur, Jaraikela and Gomlai stations estimated by five different scenarios. From Table 6, the final obtained results revealed that the proposed ML models were adequately trained and verified. The predicted outcomes during testing phase can reflect performance of the predictive models in a better way. As stated before, four hybrid SVM models and the conventional SVM model were employed for SSL prediction; every model had an equal number of MFs. From an analysis of the figure, it is clear that the considered algorithms BA, GOA, BOA, and SSA enhanced the performance of conventional SVM during training and testing stages. During the training period, SVM-SSA showed the best performance, with R^2^, RMSE, MAE, and E_NS_ values of 0.99616, 0.14994, and 0.01578, 0.99195 respectively. Similarly, during the testing period, R^2^, RMSE, MAE, and E_NS_ values for SVM-SSA are 0.97014, 15.5287, 15.3926, and 0.96481 respectively.

**Figure 7:**
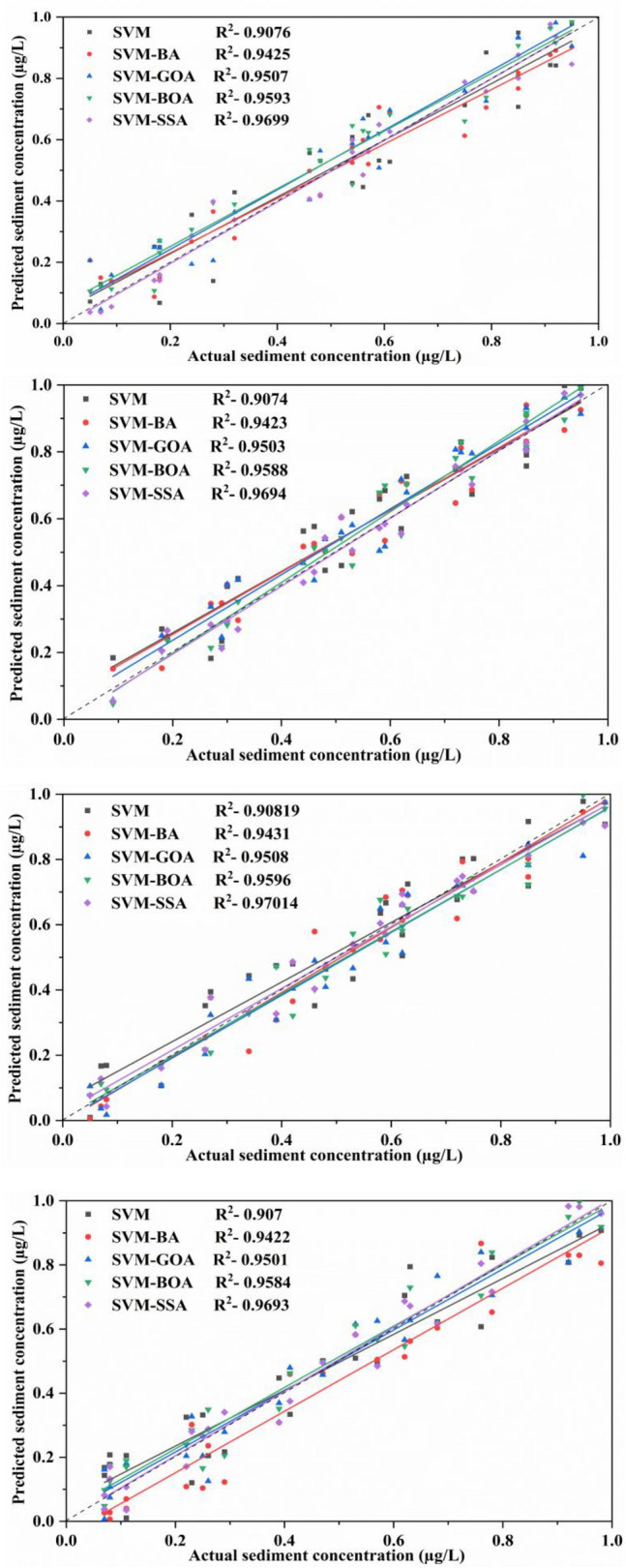
Scatter diagram of actual vs predicted SSL for best combination using five proposed models at (**a**) Tilga, (**b**) Jenapur, (**c**) Jaraikela, and (**d**) Gomlai.

The scatter plots of predicted data by SVM, SVM-BA, SVM-GOA, SVM-BOA, and SVM-SSA models against actual data during training and testing phases are reported in Fig. 7. Generally, the model shows better performance when the scatters are closer to 45° slanted line. There were differences in the regression values between the actual and predicted data for each of the recommended approaches. Scatters of SVM-SSA model are more concentrated nearby to the 45° slanted line compared to other four models. According to the graphical variations presented amid observed and predicted sediment load values; the SVM-SSA model achieved eminent correlation with maximum value for all input combinations followed by SVM-BOA, SVM-GOA, SVM-BA and ordinary SVM model. This is shown by the ability of the SVM-SSA prediction model in capturing varied sediment load observations of all four proposed gauge stations. Certainly, utilising SSA optimizer enhanced performance of SVM-SSA compared to other hybrid models and the conventional SVM model in all input scenarios.

**Figure 8:**
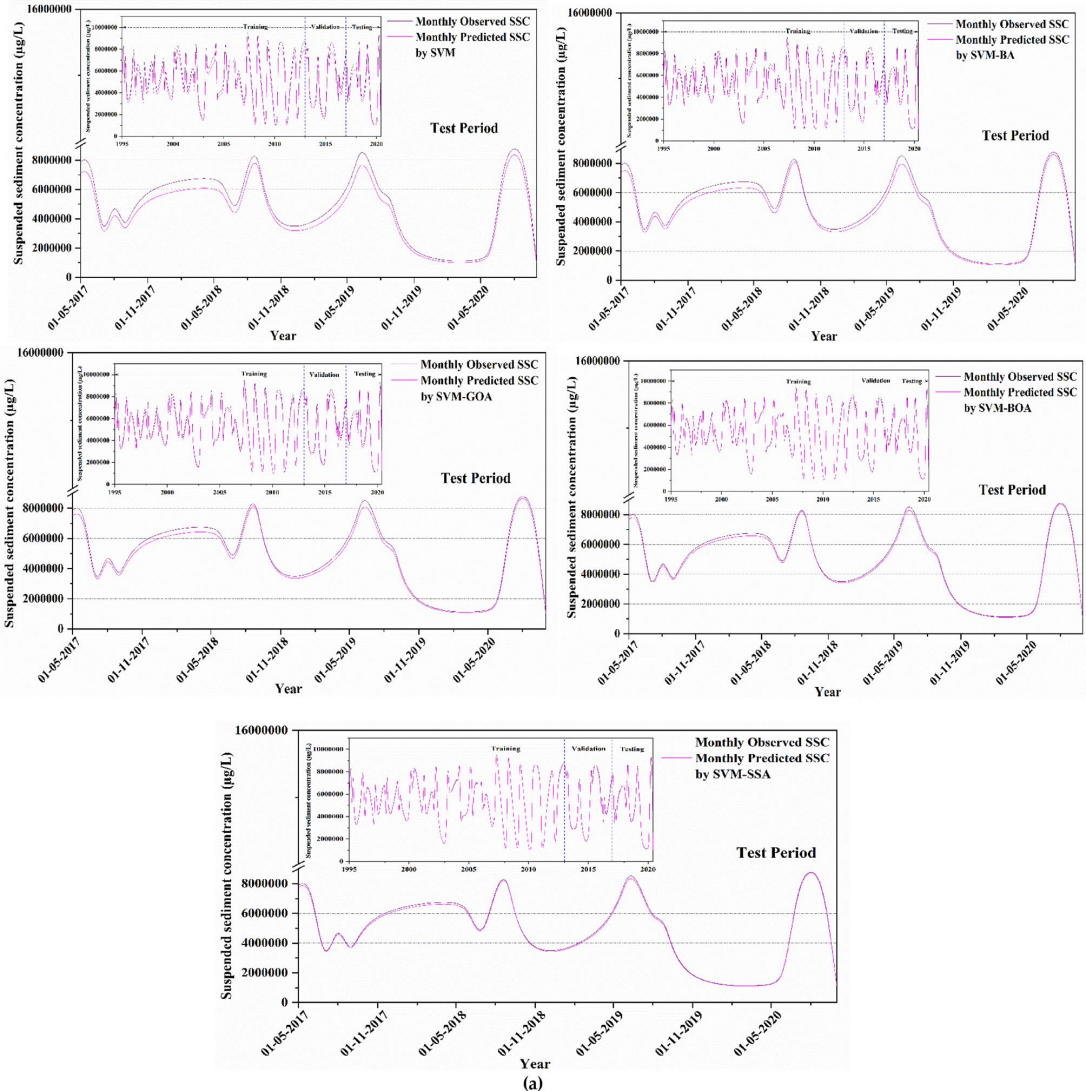

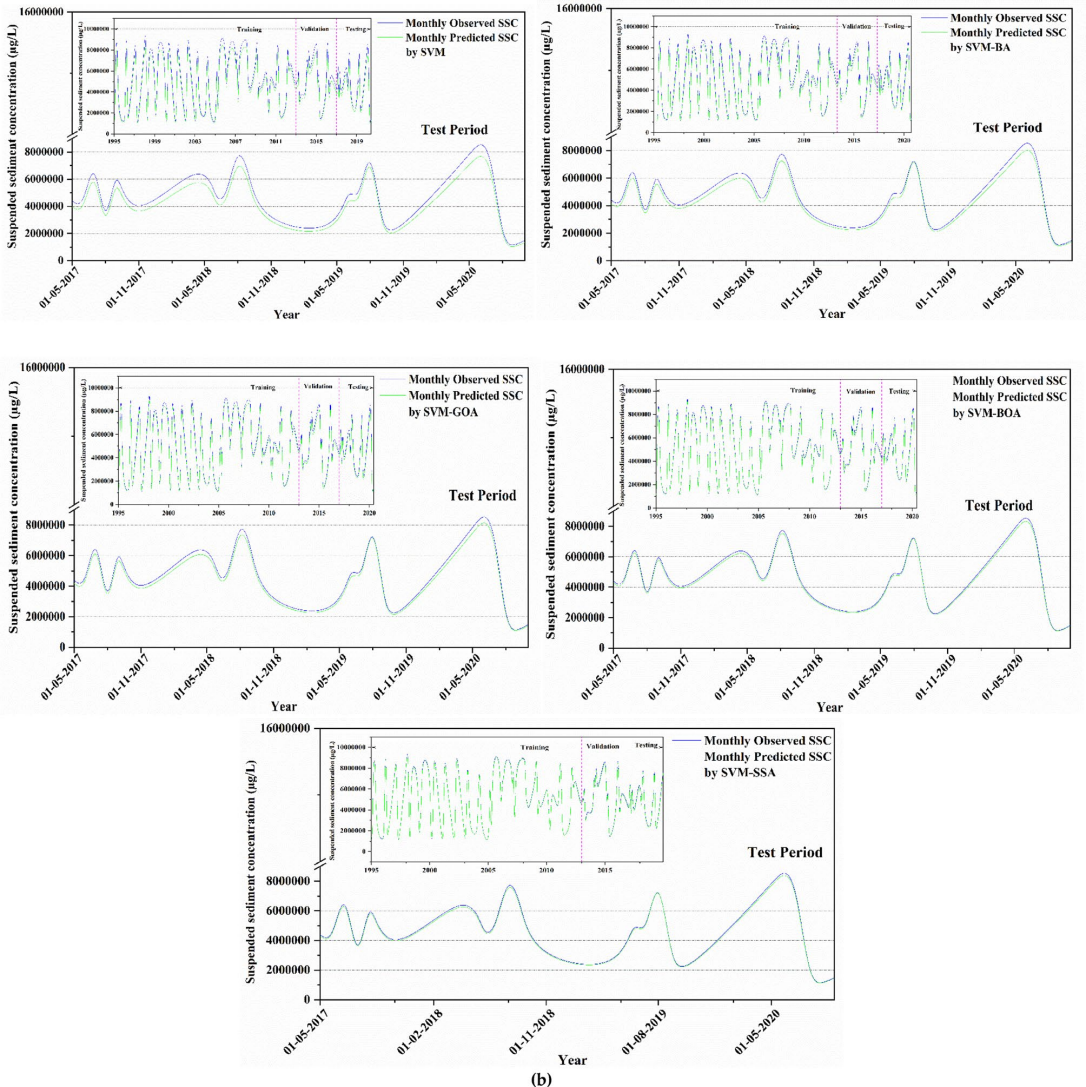

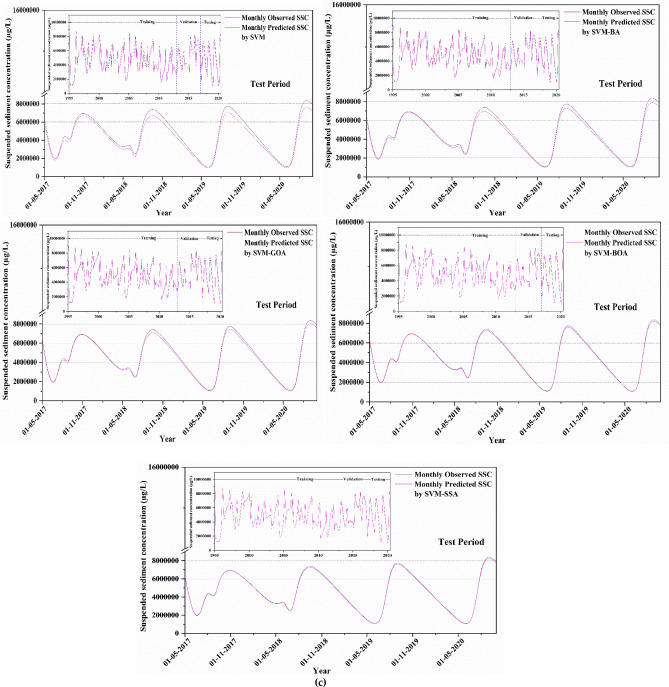

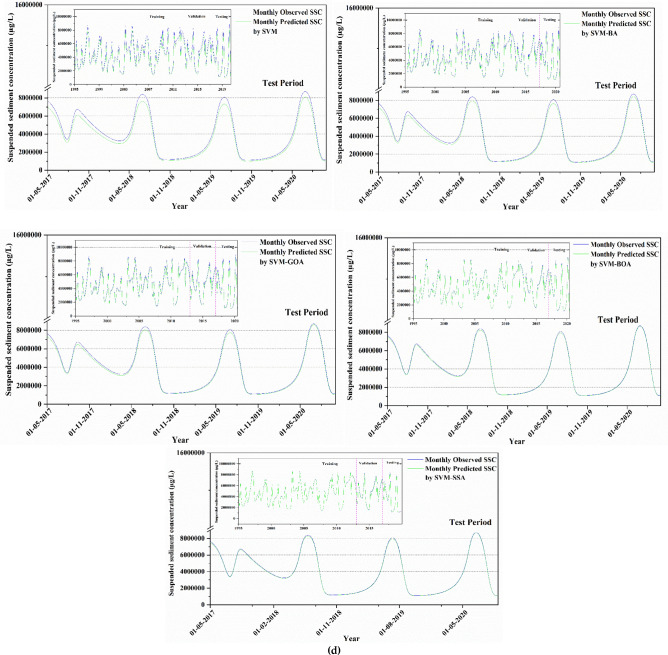
Observed vs model-predicted SSL values based on each algorithm for (**a**) Tilga, (**b**) Jenapur, (**c**) Jaraikela and (**d**) Gomlai.

To visually compare computational outcomes of SSL obtained from applied models (for best input combination) in comparison to available observed data, a time-series plot of predicted SSL data against observed values is presented in Fig. 8 for all stations. The plot illustrates that hybridized ML algorithms have superior prediction capability, predominantly in finding the peak SSL values, which is a significant development over the conventional model. As illustrated in the figure, the overall trend of SVM prediction model's predicted values can follow the fluctuation trend of the actual values to a certain degree. The prediction trend of SVM-BA and SVM-GOA models does not differ much from the each other, and the overall fluctuation from the real value is quite high. The SVM-BOA model generated prediction values with slightly less deviations from the real ones which matches with the real situation. The overall fluctuation of SVM-SSA prediction model is least having some differences in the validation phase of the trend and the actual trend with the predicted values extremely near to the real values. This figure showed that, in comparison to other predictive models, the SVM-SSA model predictions were more accurate in predicting the matching actual SSL values. Based on time series of modeled and observed SSL in Fig. 8, peak SSL data are well predicted by SVM-SSA algorithm. It can be observed from the figure that there is a minor difference amid time series of modeled and observed SSL.

**Figure 9:**
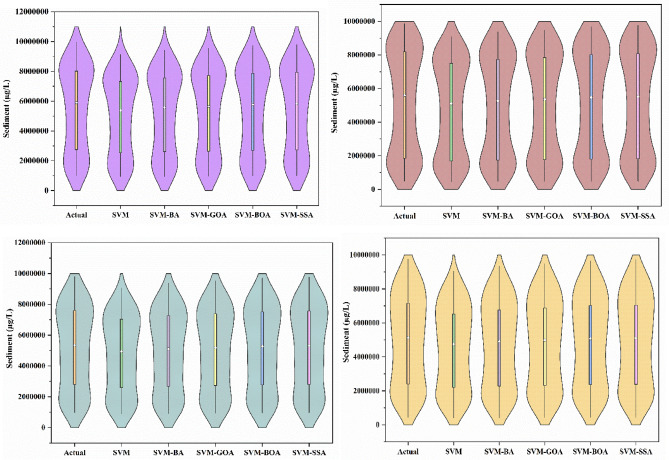
Violin plots of models for (**a**) Tilga, (**b**) Jenapur, (**c**) Jaraikela, and (**d**) Gomlai stations.

To visually evaluate performance of the models in replicating probability distribution of actual SSL data, violin plots were prepared. Violin plots of actual and model-predicted SSL data are demonstrated in Fig. 9. The similar resemblance in the form of violin signifies more likeliness of spreading observed and simulated SSL data. Figure 9 illustrates a better similarity amid actual, and SVM-SSA simulated SSL at all four sites. It was observed from the figure that the violin’s shape of hybridized models was more similar to shape of actual violin for all locations. The maximum disparity in the violin was witnessed for standalone SVM followed by SVM-BOA, SVM-GOA, and SVM-BA. An assessment of outcomes at four locations showed improved performance of all hybrid models in simulating observed SSL distributions. The reason lies is that for a long-term forecasting task, it becomes much more difficult for forecasting technique to capture dynamic change of SSL because of the more uncertain factors involved in the complex hydrological process. Therefore, the proposed method utilizing SSA to optimize parameters of SVM model can generate satisfactory forecasting outcomes. Based on different statistical indicators, performance of hybrid models was found to fluctuate slightly for different sites. Steadiness in the outcomes reveals a strong dominance of SVM-SSA model in replicating SSL in the selected study region.

**Figure 10:**
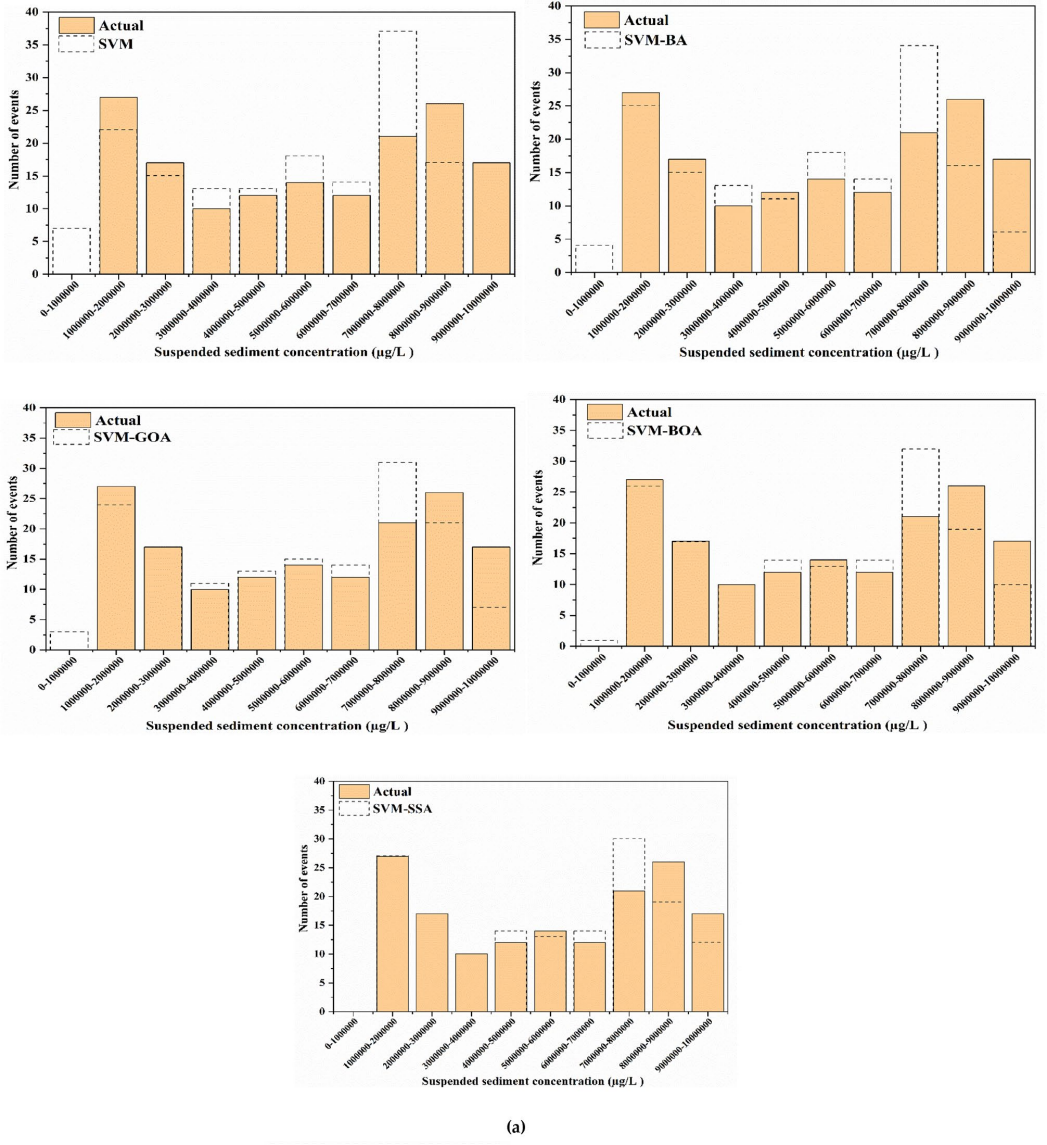

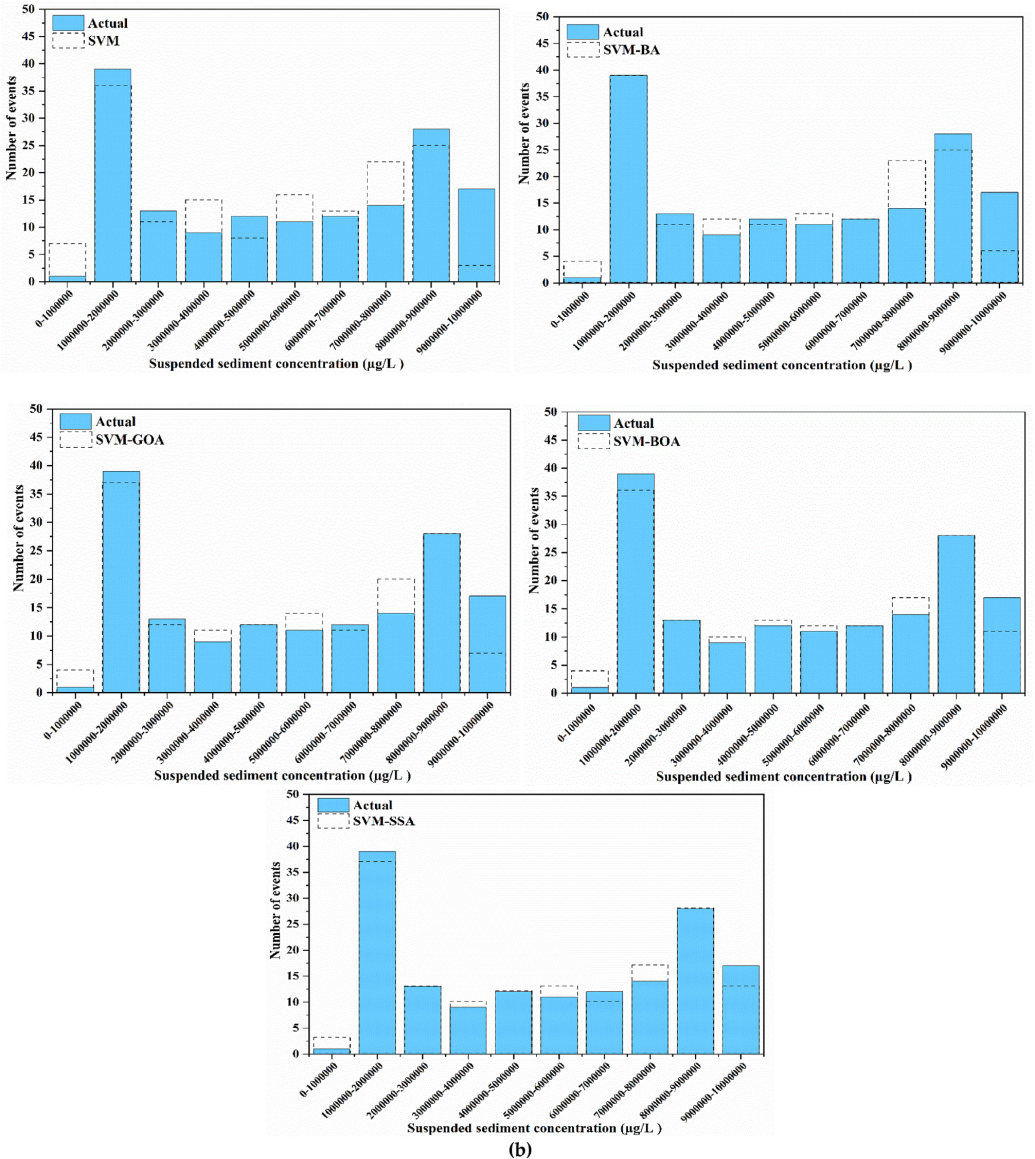

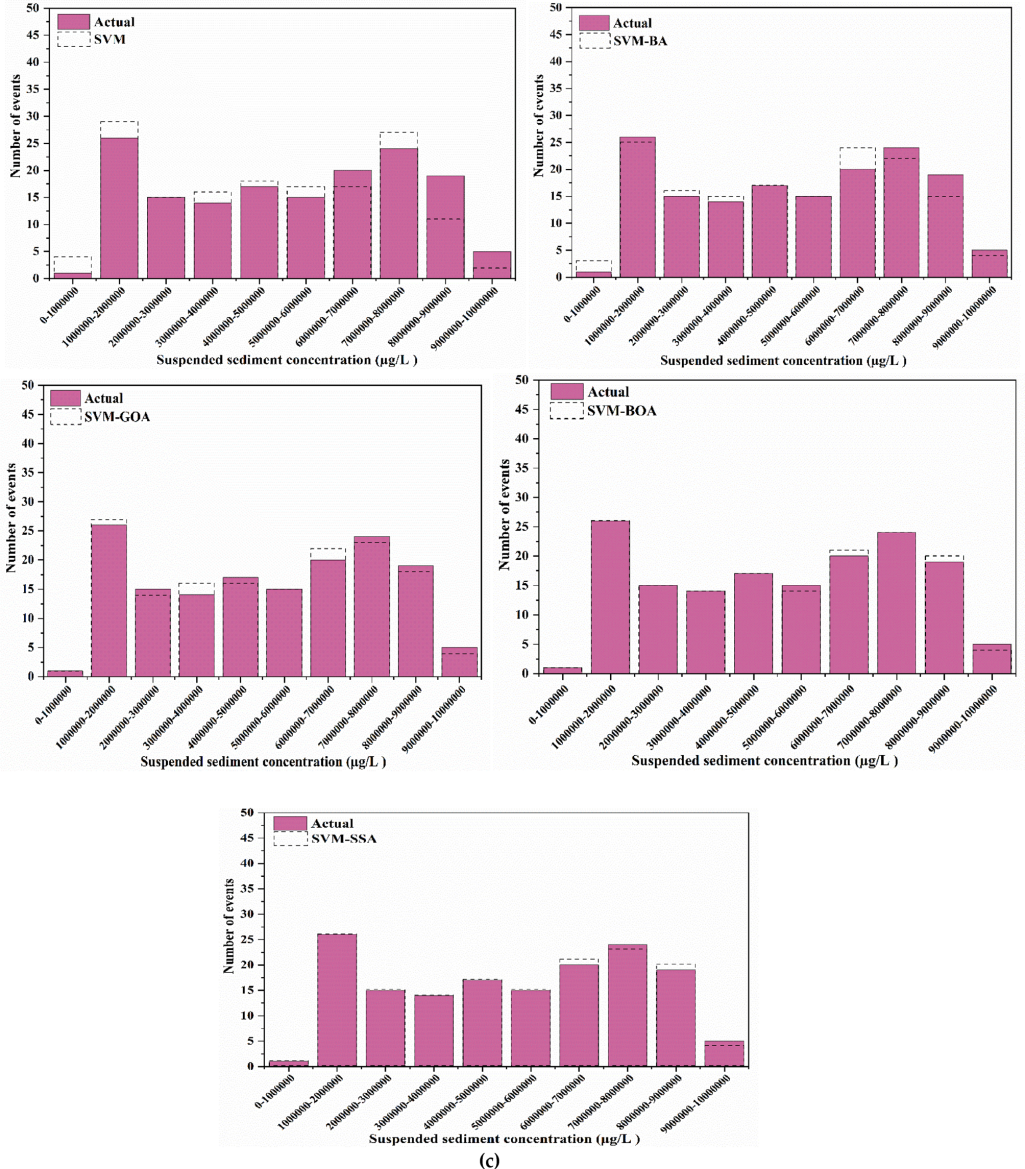

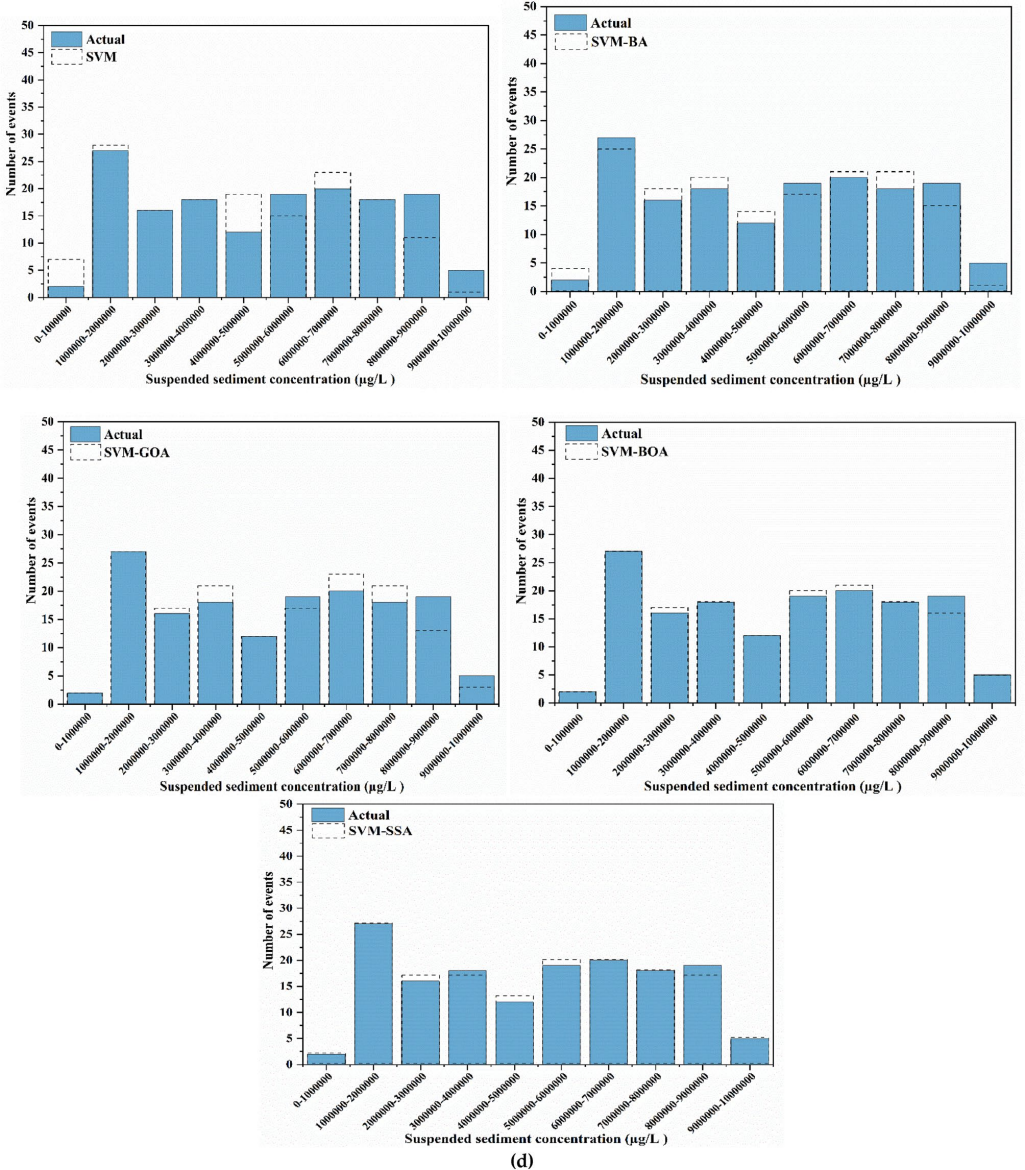
Histogram plots of best scenario in SVM-based models for SSL prediction analysis at (**a**) Tilga, (**b**) Jenapur, (**c**) Jaraikela and (**d**) Gomlai.

For a good apprehension of the estimation accuracies of five employed models, SVM, SVM-BA, SVM-GOA, SVM-BOA, and SVM-SSA, SSL values of varied series, predictions, and observations in diverse ranges are compared. Histogram plots of predicted and actual SSL values are shown in Fig. 10. Prediction of SSL at Jenapur station illustrates that for highest (100000–200000 µg/l) and lowest (300000–400000 µg/l) ranges of SSL, frequency (number of events) of precise prediction by SVM-SSA5 model displays superior agreement with frequency of actual values in comparison to frequency of precise prediction by other models. Performance of SVM-SSA, SVM-BOA, and SVM-GOA models is fairly similar in middle range values (200000–800000 µg/l); yet a slight improvement in range values is noted in performance of SVM-SSA model over SVM-BOA and SVM-GOA models whereas its performance is more enhanced than SVM-BA and SVM models. Overall, for all stations, the histogram plots show that during the training and testing periods, probability distribution of predicted values is closer to the observed values (better agreement) for the hybrid models than simple SVM model.

**Figure 11:**
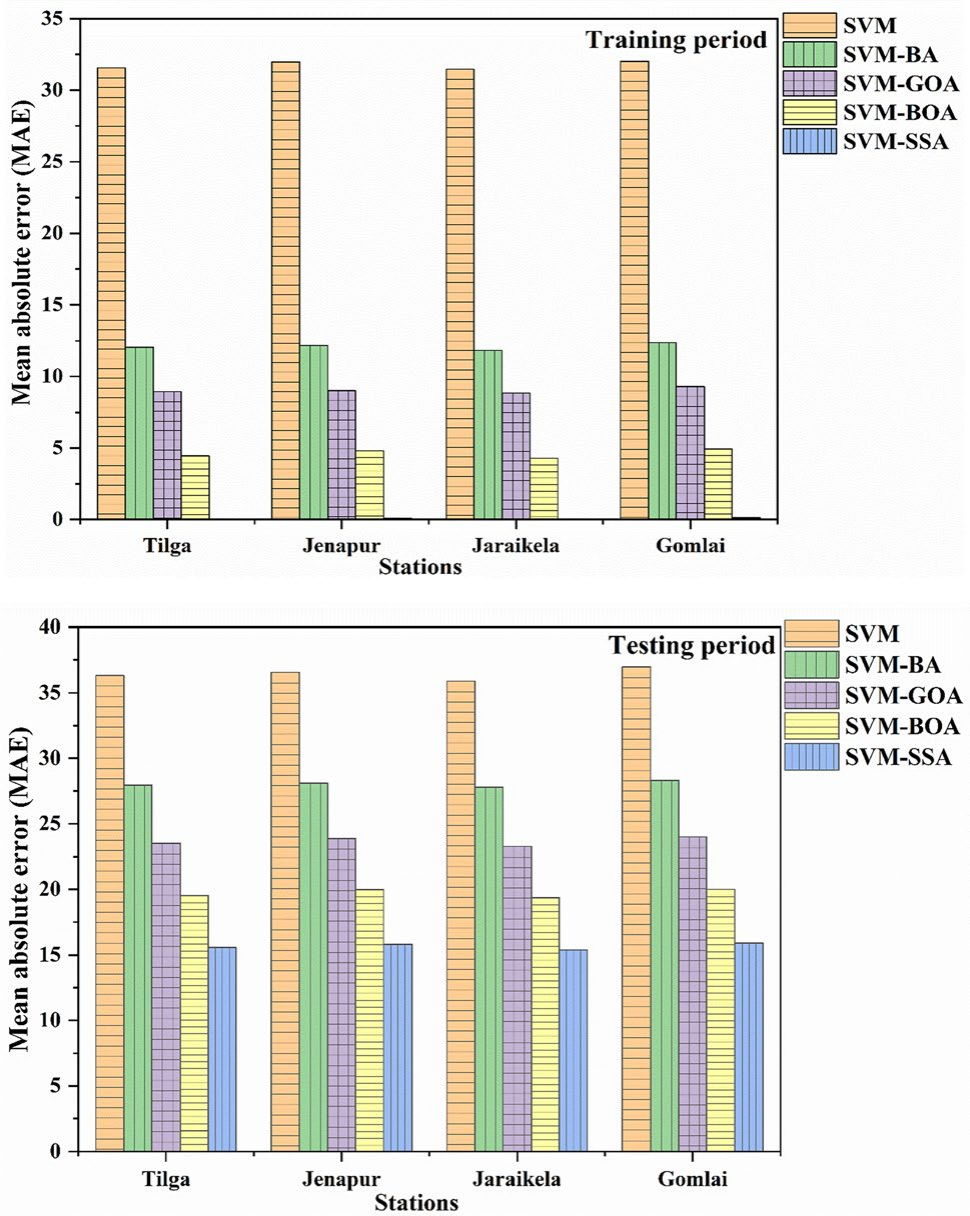
Comparison plot of MAE for (**a**) training and (**b**) testing phase.

**Figure 12:**
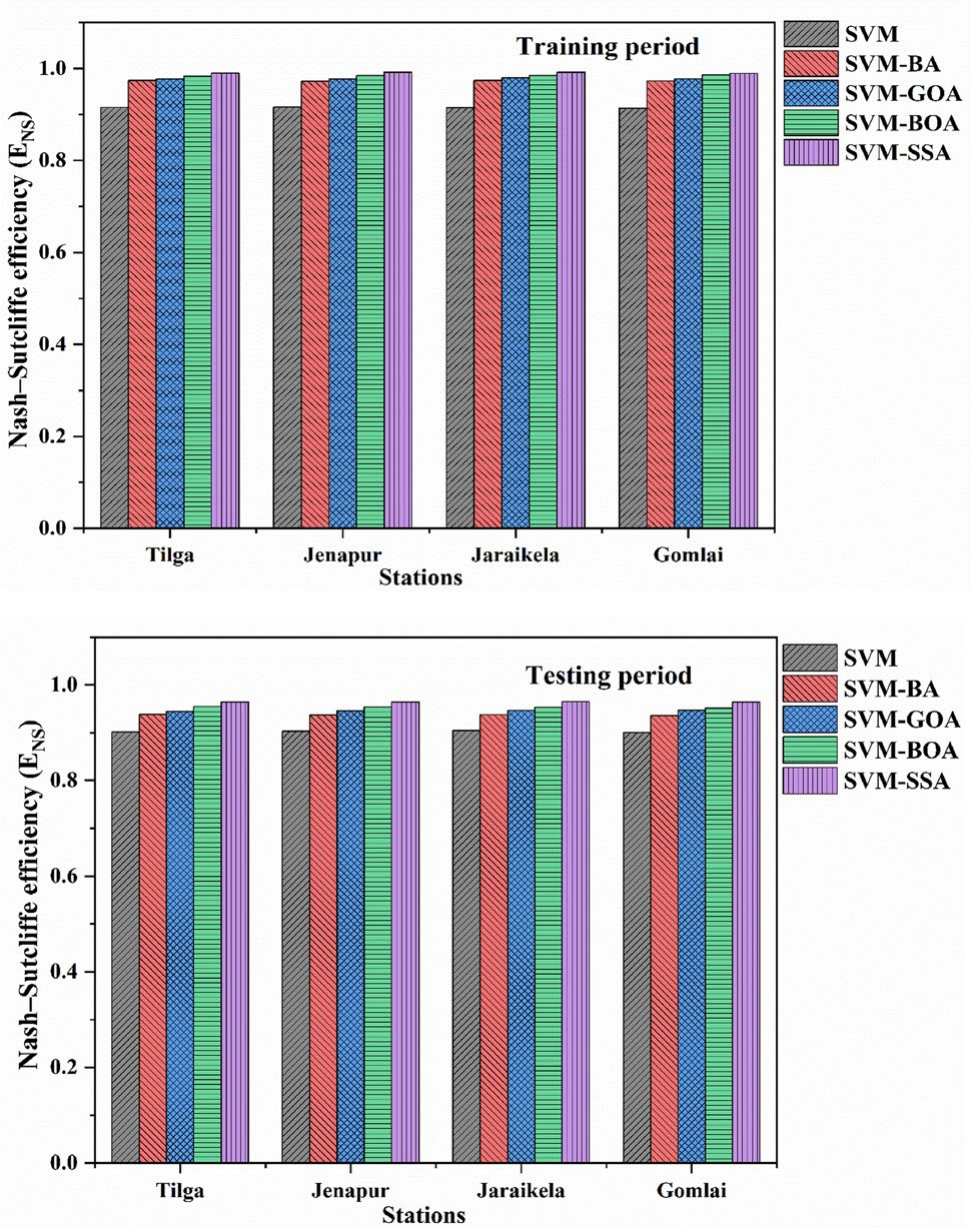
Comparison plot of E_NS_ for (**a**) training and (**b**) testing phase.

For a better representation of results obtained from the conventional and hybrid SVM models, bar charts showing MAE (mg/l) and E_NS_ are demonstrated in Figs. 11 and 12 respectively. The MAE value closer to 0 and the E_NS_ value closer to 1 imply the excellent efficacy of a model. It is clearly visible that the SVM-SSA model exhibited lower MAE values while higher E_NS_ values in the monthly forecasting scenario as compared to SVM-GOA, SVM-BOA, SVM-BA and standalone SVM models. Hence, a comparison of the outcomes shown in Figs. 11 and 12 reveals that the hybrid SVM models had better results than the conventional model. Based on both E_NS_ and MAE measures, it can be found that SVM-SSA performed superior to all other models.

A comparison of the model's ability to predict peak values in different models was conducted because, in river engineering problems, the most important portion of discharge prediction is the peak values. The time-based variations of observed vs. predicted SSL utilizing SVM-SSA, SVM-GOA, SVM-BOA, SVM-BA, and SVM algorithms are demonstrated in the form of hydrographs in Fig. 9 to assess and compare them to one another. As presented in all the presented figures, the hybrid SVM approaches indicated a better matchup with observed SSL values at all gauge stations; among which SVM-BA shows the maximum difference between the observed and estimated SSL. In contrast, the simple SVM model overestimated and underestimated certain peak values. Overall, SVM-SSA produced better SSL predictions and presented more precise estimations of the peak values than the other models.

**Figure 13:**
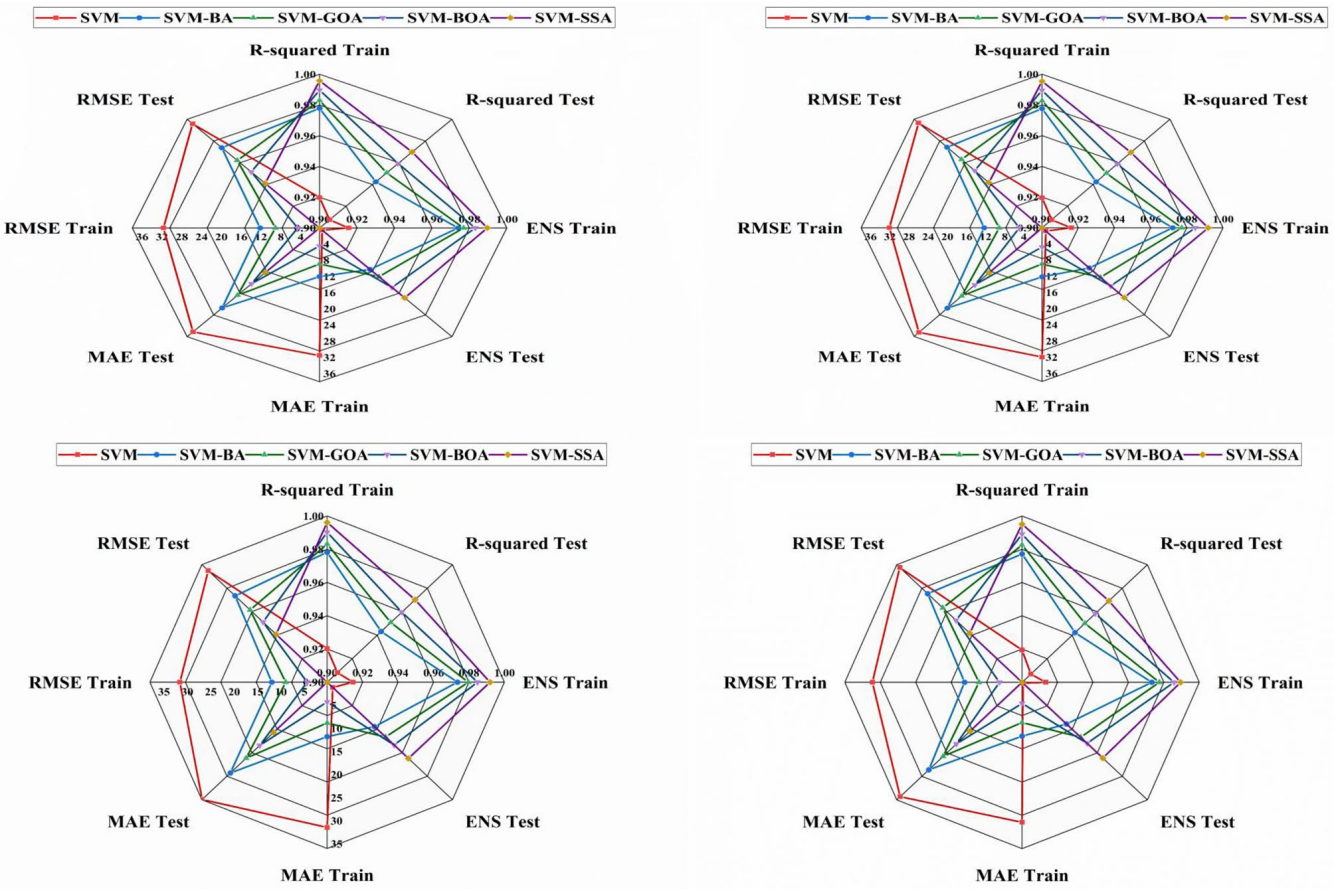
Radar chart showing RMSE, MAE, R^2^ and E_NS_ in training and testing phases of (**a**) Tilga, (**b**) Jenapur, (**c**) Jaraikela (**d**) Gomlai.

A radar chart of the performance indicators was also used to evaluate the effectiveness of the applied models (Fig. 13). The figure shows the radar charts of the metrics used for model assessments. Radar charts were utilized in various hydrological studies to provide a better diagnostic examination of the efficiency of all models ^75–78^. Each of the four statistics is displayed simultaneously on the graph. It demonstrates that the standalone SVM yields the worst results, while the SVM-SSA has the lowest RMSE and MAE, the highest ENS, and the highest R^2^. The primary benefits of using a radar chart are its ability to display the highest and lowest values of the variables in the dataset and to enable multivariable quantitative analytics.

The coefficient of determination (R^2^) values of the applied models are represented in Fig. 10. Higher R^2^ values (between 0 and 1), i.e., values close to 1, reveal a better agreement between the observed and predicted values. The figures show that the integrated SVM models had a higher value of R^2^ than conventional SVM model, and among the hybrid models, the SVM-SSA provided the closest value to 1 (R^2^ − 0.9261). It can be concluded from the R^2^ values that SVM-SSA model outperformed other models. Based on the values, the applied models are ordered as SVM-SSA, SVM-GOA, SVM-BOA, SVM-BA, and SVM.

## Discussion

Modelling of river sedimentation is one of the most complicated transformative hydrological modelling problems. Transport of suspended sediment is a dynamic non-linear system that raises significant uncertainty in characteristics of river hydrological modelling, consisting of changes in inflow and sediment load. In this context, robust methods must be employed for modelling SSL in rivers. Based on the assessment provided in previous sections, the developed SVM-SSA model effectively modeled SSL in this study, taking advantage of an optimization system to find optimum values of conventional SVM. The hybrid SVM-BOA, SVM-GOA, and SVM-BA models fail to estimate extreme SSL values accurately. However, the robust SVM-SSA model can correctly predict the maximum and minimum values with lesser errors. Based on forecasting results yielded by SVM-BA, SVM-GOA, SVM-BOA and SVM-SSA, it can be observed that there are slight differences with respect to four statistical metrics, indicating the importance of selecting an appropriate optimization algorithm for model parameter calibration. Standard SVM utilizing structural risk-minimization principle can gain good generalization performance. However, performance of SVM generally depends on optimization algorithm to calibrate parameters. Even though BA, GOA, BOA have been successfully used in solving optimization problems, all these algorithms face the drawback of easy premature convergence. As a newly proposed optimization algorithm, SSA has strong global optimum ability and can efficiently avoid local optimum issues. Hence, compared to BA, GOA and BOA, SSA affords better optimization potential.

In addition, SVM-SSA utilizes a high race optimum procedure that can learn the stochastic phenomena of SSL. It must be emphasized that field engineers are keen on using less complicated tools for practical use. Because the SVM-SSA model incorporates fewer input constraints in its architecture, it can be deliberated as an economic model for SSL prediction. The importance of this study lies in the usage of the sparrow search algorithm and its application in sediment load prediction. SSA has robust optimization capability, fast convergence speed, and broader applications than conventional heuristic search techniques. These advantages attract researchers to apply SSA for major issues like sediment load estimation, which is essential for monitoring and damage mitigation purposes. Also, high load of suspended sediment in streams are known to create unfavorable impacts on river water quality, potable water sources, reservoir or dam operations and irrigation activities.

Even though this research has made several contributions and innovations, there still exist certain drawbacks. A drawback of this research is considering a particular case study (Brahmani River basin). Further investigation will include testing the applied approach on different other streams. The authors also plan on evaluating the usage of input variables produced from weather stations utilizing numerical rainfall–runoff modeling as a substitute for input variables obtained in situ. This will facilitate the adaptation of our applied prediction models (specifically where onsite data are restricted) and probably further enhance performance of SSL estimation. Another drawback is that because of the database's incompleteness, certain possible influence aspects might not be identified. For more enhancements in stability and accuracy of predictive models, the interrelation of more robust optimization algorithms is also worth having a consideration. For future efforts, a powerful model can also attempt for solving prediction problems in several other fields of study.

## Conclusions

Prediction of river SSL is significant in planning, functioning, and preserving water structures. Sediment transport exhibits random behaviour in a river that estimates SL. This study predicted sediment loads by hybrid SVM-SSA, SVM-BOA, SVM-GOA, SVM-BA, and conventional SVM approaches. Monthly discharge and SSLs measured at Tilga, Jenapur, Jaraikela, and Gomlai stations were considered for setting up the prediction models. The prediction accuracy of the applied models is assessed utilizing statistical performance measures such as RMSE, MAE, R^2^, and E_NS_ for different arrangements of input parameters. Graphical comparisons are also used in scatter plots, time-series plots, boxplots, and histogram plots for identifying the best prediction model. Results showed that SSA is the leading algorithm with fast convergence and higher accuracy. The results indicated that best SVM-based model has the lowest MAE and RMSE values and highest E_NS_ values. This was achieved with 5 inputs after hybridization with SSA algorithm. The SVM-SSA hybrid model can precisely apprehend extreme SSL values, signifying its robustness for applicability in hydrological and water resource problems. The SVM-SSA model generated the best SSL predictions as confirmed by RMSE values of 15.6992, 15.9143, 15.5287, and 16.01885 during testing data set in Tilga, Jenapur, Jaraikela and Gomlai stations of Brahmani River. In contrast, the SVM model performed worst, as verified by the RMSE value of 36.457 (Tilga), 36.6975 (Jenapur), 36.0338 (Jaraikela), and 37.1004 (Gomlai) during testing phase. Using the proposed models to predict SSL and modelling the SSL process by taking into consideration other variables (e.g., precipitation intensity, temperature, runoff volume) can improve the present investigation. The current study is primarily relied on black-box models within the hybrid and ensemble SSL modeling framework, which is a notable limitation. Therefore, to increase the robustness of SSL modeling in future research, it is suggested for the inclusion of process-based models and optimization algorithms within the hybrid and ensemble framework. This integration of machine learning and process-based modeling approaches can potentially provide more interpretable and reliable modeling. Moreover, the authors recommend that projected SVM-SSA model could be employed in other catchments for reconfirming the model's efficiency. It is essential to employ new and excellent decomposition algorithms to improve the quality of subsequences. Furthermore, more machine learning techniques should be verified to enhance the single model forecast accuracy. As a future direction, SVM-SSA model can be verified for high time resolutions, like hourly, daily, and weekly, and combined into physical-based hydrological models.

## Data Availability

All data reported in the manuscripts are available from the corresponding author upon justified request.

## References

[1] Samadianfard S (2022). Hybrid models for suspended sediment prediction: optimized random forest and multi-layer perceptron through genetic algorithm and stochastic gradient descent methods. Neural Comput. Appl..

[2] Shadkani, S. *et al.* Comparative study of multilayer perceptron-stochastic gradient descent and gradient boosted trees for predicting daily suspended sediment load: The case study of the Mississippi River, US. *Int. J. Sediment Res.* (2020).

[3] Shojaeezadeh SA, Al-Wardy M, Nikoo MR (2024). Suspended sediment load modeling using Hydro-Climate variables and Machine learning. J. Hydrol..

[4] Boye CB, Boye P, Ziggah YY (2024). Comparative study of suspended sediment load prediction models based on artificial intelligence methods. Artif. Intell. Appl..

[5] Somura H (2012). Impact of suspended sediment and nutrient loading from land uses against water quality in the Hii River basin, Japan. J. Hydrol..

[6] Bayram A, Kankal M, Tayfur G, Önsoy H (2014). Prediction of suspended sediment concentration from water quality variables. Neural Comput. Appl..

[7] Kakaei Lafdani E, Moghaddam Nia A, Ahmadi A (2013). Daily suspended sediment load prediction using artificial neural networks and support vector machines. J. Hydrol..

[8] Nu-Fang F, Zhi-Hua S, Lu L, Cheng J (2011). Rainfall, runoff, and suspended sediment delivery relationships in a small agricultural watershed of the Three Gorges area, China. Geomorphology.

[9] Sadeghi SHR, Mostafazadeh R (2016). Triple diagram models for changeability evaluation of precipitation and flow discharge for suspended sediment load in different time scales. Environ. Earth Sci..

[10] Bathrellos GD, Skilodimou HD, Chousianitis K, Youssef AM, Pradhan B (2017). Suitability estimation for urban development using multi-hazard assessment map. Sci. Total Environ..

[11] Ali G, Abbas S (2013). Exploring CO_2_ sources and sinks nexus through integrated approach: Insight from Pakistan. J. Environ. Inform..

[12] Cigizoglu HK (2004). Estimation and forecasting of daily suspended sediment data by multi-layer perceptrons. Adv. Water Resour..

[13] Nourani V (2009). Using artificial neural networks (ANNs) for sediment load forecasting of Talkherood river mouth. J. Urban Environ. Eng..

[14] Sahoo, A., Behera, S. & Sharma, N. Performance comparison of LS-SVM and ELM-based models for precipitation prediction in Barak valley: A case study. In *International conference on advances in communication technology and computer engineering.*10.1063/5.0132387

[15] Samantaray S, Sahoo P, Sahoo A, Satapathy DP (2023). Flood discharge prediction using improved ANFIS model combined with hybrid particle swarm optimisation and slime mould algorithm. Environ. Sci. Pollut. Res..

[16] Samantaray S, Sahoo A (2023). Prediction of flow discharge in Mahanadi River Basin, India, based on novel hybrid SVM approaches. Environ. Dev. Sustain..

[17] Samantaray S, Das SS, Sahoo A, Satapathy DP (2022). Evaluating the application of metaheuristic approaches for flood simulation using GIS: A case study of Baitarani river Basin, India. Mater. Today Proc..

[18] Achite M, Yaseen ZM, Heddam S, Malik A, Kisi O (2022). Advanced machine learning models development for suspended sediment prediction: Comparative analysis study. Geocarto Int..

[19] Adnan RM (2021). Predictability performance enhancement for suspended sediment in rivers: Inspection of newly developed hybrid adaptive neuro-fuzzy system model. Int. J. Sediment Res..

[20] Kisi O, Yaseen ZM (2019). The potential of hybrid evolutionary fuzzy intelligence model for suspended sediment concentration prediction. Catena.

[21] Nourani V, Alizadeh F, Roushangar K (2016). Evaluation of a two-stage SVM and spatial statistics methods for modeling monthly river suspended sediment load. Water Resour. Manag..

[22] Yaseen ZM (2023). A new benchmark on machine learning methodologies for hydrological processes modelling: A comprehensive review for limitations and future research directions. Knowl.-Based Eng. Sci..

[23] Tayfur G (2002). Artificial neural networks for sheet sediment transport. Hydrol. Sci. J..

[24] Afan HA, El-shafie A, Mohtar WHMW, Yaseen ZM (2016). Past, present and prospect of an Artificial Intelligence (AI) based model for sediment transport prediction. J. Hydrol..

[25] Boukhrissa ZA, Khanchoul K, Le Bissonnais Y, Tourki M (2013). Prediction of sediment load by sediment rating curve and neural network (ANN) in El Kebir catchment, Algeria. J. Earth Syst. Sci..

[26] Azamathulla HM, Ghani AA, Chang CK, Hasan ZA, Zakaria NA (2010). Machine learning approach to predict sediment load - A case study. Clean Soil Air Water.

[27] Azamathulla HM, Cuan YC, Ghani AA, Chang CK (2012). Suspended sediment load prediction of river systems: GEP approach. Arab. J. Geosci..

[28] Olyaie E, Banejad H, Chau K-W, Melesse AM (2015). A comparison of various artificial intelligence approaches performance for estimating suspended sediment load of river systems: a case study in United States. Environ. Monit. Assess..

[29] Kaveh K, Kaveh H, Bui MD, Rutschmann P (2020). Long short-term memory for predicting daily suspended sediment concentration. Eng. Comput..

[30] AlDahoul N (2021). Suspended sediment load prediction using long short-term memory neural network. Sci. Rep..

[31] Rezaei K, Pradhan B, Vadiati M, Nadiri AA (2021). Suspended sediment load prediction using artificial intelligence techniques: Comparison between four state-of-the-art artificial neural network techniques. Arab. J. Geosci..

[32] Kumar A, Tripathi VK (2022). Capability assessment of conventional and data-driven models for prediction of suspended sediment load. Environ. Sci. Pollut. Res..

[33] Tao H (2021). Artificial intelligence models for suspended river sediment prediction: state-of-the art, modeling framework appraisal, and proposed future research directions. Eng. Appl. Comput. Fluid Mech..

[34] Bandini F (2020). Unmanned Aerial System (UAS) observations of water surface elevation in a small stream: Comparison of radar altimetry, LIDAR and photogrammetry techniques. Remote Sens. Environ..

[35] Asadi M, Fathzadeh A, Kerry R, Ebrahimi-Khusfi Z, Taghizadeh-Mehrjardi R (2021). Prediction of river suspended sediment load using machine learning models and geo-morphometric parameters. Arab. J. Geosci..

[36] Ebtehaj I, Bonakdari H, Sharifi A (2014). Design criteria for sediment transport in sewers based on self-cleansing concept. J. Zhejiang Univ. Sci. A.

[37] Goldstein EB, Coco G, Plant NG (2019). A review of machine learning applications to coastal sediment transport and morphodynamics. Earth-Sci. Rev..

[38] Tikhamarine Y, Souag-Gamane D, Ahmed AN, Kisi O, El-Shafie A (2020). Improving artificial intelligence models accuracy for monthly streamflow forecasting using grey Wolf optimization (GWO) algorithm. J. Hydrol..

[39] Valikhan-Anaraki M (2019). Development of a novel hybrid optimization Algorithm for minimizing irrigation deficiencies. Sustainability.

[40] Sahoo BB, Sankalp S, Kisi O (2023). A novel smoothing-based deep learning time-series approach for daily suspended sediment load prediction. Water Resour. Manag..

[41] Sahoo BB, Jha R, Singh A, Kumar D (2019). Application of support vector regression for modeling low flow time series. KSCE J. Civ. Eng..

[42] Banadkooki FB (2020). Enhancement of groundwater-level prediction using an integrated machine learning model optimized by whale algorithm. Nat. Resour. Res..

[43] Abba SI (2020). Implementation of data intelligence models coupled with ensemble machine learning for prediction of water quality index. Environ. Sci. Pollut. Res..

[44] Afan HA (2020). Input attributes optimization using the feasibility of genetic nature inspired algorithm: Application of river flow forecasting. Sci. Rep..

[45] Ehteram M (2019). Investigation on the potential to integrate different artificial intelligence models with Metaheuristic algorithms for improving river suspended sediment predictions. Appl. Sci..

[46] Yousif AA (2019). Open channel sluice gate scouring parameters prediction: Different scenarios of dimensional and non-dimensional input parameters. Water.

[47] Ehteram M (2018). Reservoir operation by a new evolutionary algorithm: Kidney algorithm. Water Resour. Manag..

[48] Farzin S (2018). Flood routing in river reaches using a three-parameter Muskingum model coupled with an improved bat algorithm. Water.

[49] Allawi MF, Jaafar O, Ehteram M, Mohamad Hamzah F, El-Shafie A (2018). Synchronizing Artificial Intelligence models for operating the dam and reservoir system. Water Resour. Manag..

[50] Ahmed MM, Houssein EH, Hassanien AE, Taha A, Hassanien E (2019). Maximizing lifetime of large-scale wireless sensor networks using multi-objective whale optimization algorithm. Telecommun. Syst..

[51] Yahya NA, Samsudin R, Shabri A, Saeed F (2019). Combined group method of data handling models using artificial bee colony algorithm in time series forecasting. Proc. Comput. Sci..

[52] Rajaee T (2011). Wavelet and ANN combination model for prediction of daily suspended sediment load in rivers. Sci. Total Environ..

[53] Kisi O, Docheshmeh Gorgij A, Zounemat-Kermani M, Mahdavi-Meymand A, Kim S (2019). Drought forecasting using novel heuristic methods in a semi-arid environment. J. Hydrol..

[54] Adnan RM, Liang Z, El-Shafie A, Zounemat-Kermani M, Kisi O (2019). Prediction of suspended sediment load using data-driven models. Water.

[55] Hassanpour F, Sharifazari S, Ahmadaali K, Mohammadi S, Sheikhalipour Z (2019). Development of the FCM-SVR hybrid model for estimating the suspended sediment load. KSCE J. Civ. Eng..

[56] Ehteram M (2020). Design of a hybrid ANN multi-objective whale algorithm for suspended sediment load prediction. Environ. Sci. Pollut. Res..

[57] Nhu V-H (2020). Monthly suspended sediment load prediction using artificial intelligence: Testing of a new random subspace method. Hydrol. Sci. J..

[58] Zounemat-Kermani M, Mahdavi-Meymand A, Alizamir M, Adarsh S, MundherYaseen Z (2020). On the complexities of sediment load modeling using integrative machine learning: An application to the great river of Loíza in Puerto Rico. J. Hydrol..

[59] Farzin S, Valikhan Anaraki M (2021). Modeling and predicting suspended sediment load under climate change conditions: A new hybridization strategy. J. Water Clim. Chang..

[60] Vapnik V, Guyon I, Hastie T (1995). Support vector machines. Mach. Learn.

[61] Jamei M (2022). Designing a multi-stage expert system for daily ocean wave energy forecasting: A multivariate data decomposition-based approach. Appl. Energy.

[62] Doroudi S, Sharafati A, Mohajeri SH (2021). Estimation of daily suspended sediment load using a novel hybrid support vector regression model incorporated with observer-teacher-learner-based optimization method. Complexity.

[63] Yang, X. A new metaheuristic bat-inspired algorithm. *Coop. Strateg. Optim. (NICSO 2010)* (2010).

[64] Essa KS, Diab ZE (2022). Magnetic data interpretation for 2D dikes by the metaheuristic bat algorithm: Sustainable development cases. Sci. Rep..

[65] Saremi S, Mirjalili S, Lewis A (2017). Grasshopper optimisation algorithm: Theory and application. Adv. Eng. Softw..

[66] Qin P, Hu H, Yang Z (2021). The improved grasshopper optimization algorithm and its applications. Sci. Rep..

[67] Ewees AA, Abd Elaziz M, Houssein EH (2018). Improved grasshopper optimization algorithm using opposition-based learning. Expert Syst. Appl..

[68] Barman M, Dev Choudhury NB, Sutradhar S (2018). A regional hybrid GOA-SVM model based on similar day approach for short-term load forecasting in Assam, India. Energy.

[69] Arora S, Singh S (2018). Butterfly optimization algorithm: A novel approach for global optimization. Soft Comput..

[70] Zhang X, Liu F, Yin Q, Qi Y, Sun S (2023). A runoff prediction method based on hyperparameter optimisation of a kernel extreme learning machine with multi-step decomposition. Sci. Rep..

[71] Xue J, Shen B (2020). A novel swarm intelligence optimization approach: Sparrow search algorithm. Syst. Sci. Control Eng..

[72] Liu R (2022). Spatial prediction of groundwater potentiality using machine learning methods with Grey Wolf and Sparrow Search Algorithms. J. Hydrol..

[73] Bhattarai A, Qadir D, Sunusi AM, Getachew B, Mallah AR (2023). Dynamic sliding window-based long short-term memory model development for pan evaporation forecasting. Knowl.-Based Eng. Sci..

[74] Elsayed S (2023). Interpretation the influence of hydrometeorological variables on soil temperature prediction using the potential of deep learning model. Knowl.-Based Eng. Sci..

[75] Kushwaha NL (2021). Data intelligence model and meta-heuristic algorithms-based pan evaporation modelling in two different agro-climatic zones: A case study from northern India. Atmosphere.

[76] Granata F, Di Nunno F (2021). Forecasting evapotranspiration in different climates using ensembles of recurrent neural networks. Agric. Water Manag..

[77] Asnake Metekia W, Garba Usman A, Hatice Ulusoy B, Isah Abba S, Chirkena Bali K (2022). Artificial intelligence-based approaches for modeling the effects of spirulina growth mediums on total phenolic compounds. Saudi J. Biol. Sci..

[78] Khosravi K, Golkarian A, Melesse AM, Deo RC (2022). Suspended sediment load modeling using advanced hybrid rotation forest based elastic network approach. J. Hydrol..

